# Exploring the role of EMT in ovarian cancer progression using a multiscale mathematical model

**DOI:** 10.1038/s41540-025-00508-y

**Published:** 2025-04-17

**Authors:** Samuel Oliver, Michael Williams, Mohit Kumar Jolly, Deyarina Gonzalez, Gibin Powathil

**Affiliations:** 1 Department of Mathematics, Swansea University, Swansea, UK; 2 Department of Biomedical Sciences, Swansea University, Swansea, UK; 3 Department of Bioengineering, Indian Institute of Science, Bangalore, India

**Keywords:** Cancer, Cell biology, Applied mathematics

## Abstract

Epithelial-to-mesenchymal transition (EMT) plays a key role in the progression of cancer tumours, significantly reducing the success of treatment. EMT occurs when a cell undergoes phenotypical changes, resulting in enhanced drug resistance, higher cell plasticity, and increased metastatic abilities. Here, we employ a 3D agent-based multiscale modelling framework using PhysiCell to explore the role of EMT over time in two cell lines, OVCAR-3 and SKOV-3. This approach allows us to investigate the spatiotemporal progression of ovarian cancer and the impacts of the conditions in the microenvironment. OVCAR-3 and SKOV-3 cell lines possess highly contrasting tumour layouts, allowing a wide range of different tumour dynamics and morphologies to be tested and studied. Along with performing sensitivity analysis on the model, simulation results capture the biological observations and trends seen in tumour growth and development, thus helping to obtain further insights into OVCAR-3 and SKOV-3 cell line dynamics.

## Introduction

Epithelial-to-mesenchymal transition (EMT) is a process in which epithelial cells undergo phenotypic changes, enabling a reduction in cell-cell adhesion and enhancing migratory abilities^1,2^. EMT is essential for normal tissue functionality within the body^3^. It allows the closure of developmental neural tubes^4^, plays a key involvement in embryogenesis^5^, and enables wound healing to occur^6^. Despite the reliance of the human body on this process, the role of EMT can occasionally become detrimental and further complicate treatment for diseases. EMT is heavily linked to cystic fibrosis by causing goblet cell and pneumocyte hyperplasia in the lungs^7^. Rheumatic diseases have also been linked to EMT, with rheumatoid joints expressing an abundant amount of transforming growth factor beta (TGF-*β*) in the synovial fluid^8,9^.

Since EMT plays a large role in many different processes, it was recently suggested to separate EMT into three main types^10,11^. EMT occurring during a self-contained process requiring multiple cell types to be generated such as organ development and embryo formation is classified as type 1. EMT associated with repair such as wound healing, organ fibrosis, and tissue regeneration is classified as type 2. This repair discontinues upon completion and when inflammation is reduced. The third type of EMT includes instances where there is a genetic and epigenetic difference between the epithelial and mesenchymal cell types. Type 3 is the key type of EMT associated with cancer progression and metastasis.

Instead of being considered a binary switch, EMT is now considered to be a more continuous procedure^12^. Cells can fluctuate through a multi-step process during which they may show partial epithelial and mesenchymal characteristics^13,14^. This leads to a more complex differentiation process between classifications of cells. Various biological markers are used to conclude the placement of these cells along the EMT scale^15^. E-cadherin is a surface marker used for the identification of epithelial cells^16^, while N-cadherin, vimentin, and fibronectin are markers to locate mesenchymal cells^17^. The ratio of these markers can be used to conclude the epithelial/mesenchymal balance determined for each individual cell^18^.

EMT is a crucial step in cancer progression^19^, allowing mesenchymal cells within a tumour to have lower cell-cell adhesion forces due to a reduced E-cadherin expression on the cell surface^20^. This allows the cells to break away from the main tumour location and escape from the brick-like structure they were previously a part of ^21^. This relocation of cells can cause metastasis away from the primary tumour site. Metastatic cases are responsible for over 90% of all cancer-related deaths^22^. One justification for this is the improved drug resistance possessed by the slower-cycling metastatic cells^23,24^. These metastatic cells can obtain resistance to anoikis, a type of programmed cell death caused by a detachment from the surrounding extracellular matrix^25^. Cells can also switch from a phenotype tailored for proliferation to a phenotype which targets invasion around the body^26^. This lack of proliferation hinders the effectiveness of the drug, as targeting the rapidly dividing cells is no longer efficacious^27,28^. This effect is responsible for lower long-term treatment dosages occasionally being beneficial. Higher dosages can eradicate the susceptible, less concerning epithelial cells, therefore making space and freeing up resources for the mesenchymal cells to exploit^29–31^. This paper will focus on the link between the tumour microenvironment and the initialization of EMT within cells^32,33^.

As well as the composition, the overall size of the tumour has a major impact on the likelihood of successful treatment for cancer patients. The rapid growth of cancer tumours is mainly due to the increased rate at which cancer cells can divide and multiply^34^. Cancer cells have a higher proliferation rate to that of normal cells, gaining an ability to bypass various cell cycling checkpoints^35^. The cell cycle is split into four stages: gap 1 (G1), synthesis (S), gap 2 (G2), and mitosis (M)^36^. Three main DNA damage checkpoints are present throughout the cycle, located at the end of the G1, S, and G2 phases to ensure damage cannot be passed onto future generations^37^. Along side these, an antephase checkpoint ensures that environmental conditions such as oxygen levels and available surrounding space are favourable to support attempting cell division^38^. When arriving at a checkpoint, either the cell passes the checkpoint and continues the journey through the cell cycle, or fails at the checkpoint at which point the cell may initiate cell death or enter quiescence, a state of reversible exit from the cell cycle^39^. The ability of cancer cells to avoid these checkpoints allows the cells to complete steps of the cycle faster and with higher chances of success, causing the tumours to form and progress so rapidly.

It has been shown that cells are also capable of undergoing mesenchymal-to-epithelial transition (MET)^40,41^. This is the reverse process of EMT, where mesenchymal cells transition back to epithelial cells and regain the epithelial phenotype and behaviours previously exhibited^42–44^. The phenomenon of MET is primarily seen in relocated mesenchymal cells which return to focusing on proliferation^45^. While completing EMT allows a cell to travel with more ease throughout the body, MET enables transformation back to the faster proliferating, more stable epithelial phenotype^46,47^. This allows the tumour to grow faster and spread throughout the body more rapidly^48,49^.

With a higher focus on the role of EMT in recent years^50,51^, mathematical models are becoming a valuable asset to scientific research into improving survival and cancer treatment success rates^52–54^. In-silico models offer a fast and accurate alternative to in-vivo or in-vitro models^55^, both of which expend physical resources such as the cell lines needed to perform the experiments, time required for the biological processes to complete, and potentially the need for complex ethical and moral justifications^56,57^. These models can be used to test the impacts of different parameter values or conditions in the microenvironment by using simple adaptations, eventually informing experimental settings^58^.

EMT has become an increasingly popular area for mathematical modelling, with many various approaches taken in the last decade. MacLean et al.^59^ use an ODE model to measure the population sizes of two cell types: epithelial and mesenchymal. A reversible binary switch is assumed to occur between the cell types, with switch rates dependent on the total population sizes of each. Franssen et al.^60^ later models the metastatic spread of cancer using a non-binary classification of the EMT process. Partial EMT states are introduced and use an agent-based model with a system of ordinary and partial differential equations within a 2D domain. The model allows cell detachment in partial EMT mesenchymal cells, with cells primarily around the tumour periphery undergoing transition to break away from the primary tumour. Other models such as those produced by He et al.^61^ and Mooney et al.^62^ include feedback loops of various transcription factors. Both the EMT and MET processes are modelled using systems of ODEs, with the correlations between the model output and various input parameters such as gene expressions studied. Murphy et al.^63^ develop both discrete and continuum mathematical models, inducing EMT and cell detachment through chemical signal concentrations. These concentrations also affect the proliferation rate and size of cells in the models. An extensive review performed by Jolly et al.^64^ has shown how past mathematical models have helped improve the understanding of EMT in cancer cells. The reverse process, MET, is included in the review to explore how the plasticity of the cells along this scale can impact the models and their findings.

Here, we investigate the role of EMT in the progression of ovarian cancer by developing a multiscale mathematical and computational model with a focus on two common ovarian cancer cell lines: OVCAR-3, a human ovarian epithelial carcinoma cell line^65^ and SKOV-3, a human ovarian adenocarcinoma cell line^66^. This model allows cells to migrate through the domain, proliferate at microenvironment-dependent rates, and progress through EMT in a biologically realistic manner. The model is based on experimental data and processes found in past literature. During the simulations, information on each cell such as position, velocity, surrounding oxygen levels, and cell cycle stage can be tracked and analysed.

The model will also be used to study the importance of including MET in tumour dynamics, a key process when modelling metastatic cancers. Direct comparisons will be made between simulations both including and excluding the presence of MET. Sensitivity analysis on the key parameters is performed to help quantify the role of EMT and its association with tumour size and composition over time. Following model validation using experimental data, future predictions are made on different initial states of the tumour. Temporal dynamics of tumours initialized with epithelial and mesenchymal cells across different cell lines are compared, with the importance of accurately distinguishing the cell lines highlighted. By investigating the progression of cancer tumours in various scenarios, we aim to make predictions for how cell populations and tumour compositions are likely to change with time. Given these results, future adaptations of this model may be used to incorporate various treatment plans. Comparing these plans can provide insights into optimal treatment protocols. Ideally, patients diagnosed at a certain stage could have their tumour recreated as a digital twin and simulated using this model, incorporating this treatment method to provide the best prognosis possible.

## Results

Here, we study temporal tumour evolution over 96 h of simulated time using a mathematical model. OVCAR-3 and SKOV-3 epithelial cells are placed in the domain, with the cross-sections of the tumour shown each day until the completion of day four. The simulation results are then compared to the biological observations and data obtained from the in-vitro experiments.

**Fig. 1:**
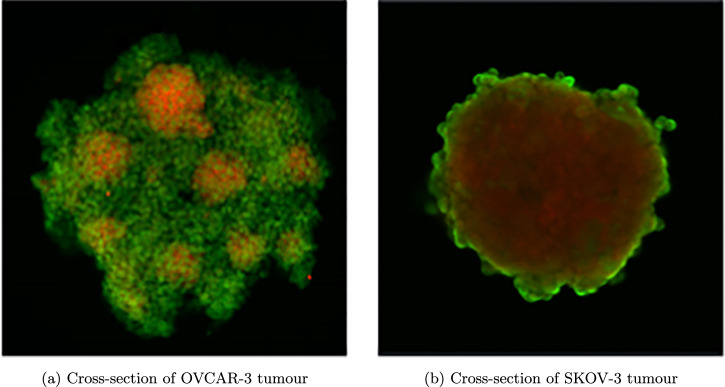
Cross-section of tumours upon biological experiment completion. Green areas represent high levels of E-cadherin, expressed in epithelial cells. Red areas show high expression of N-cadherin, a marker for mesenchymal cells. **a** Small clumps of mesenchymal cells are shown in OVCAR-3 tumours surrounded by a backdrop of epithelial cells. **b** Mesenchymal cells make up the majority of SKOV-3 tumours, with a thin layer of epithelial cells appearing on the periphery. Details on the experimental conditions can be found in Section “Experimental Methods”.

The two cell lines used here to investigate the EMT process possess highly contrasting characteristics, making the mathematical model more versatile and adaptable to cell lines. Figure 1 shows the cross-section of (a) OVCAR-3 and (b) SKOV-3 3D tumour spheroids following an in-vitro experiment. SKOV-3 cells are seen to show a high expression of N-cadherin (red), suggesting a large mesenchymal cell population within the tumour^67^. During biological observations of SKOV-3 spheroid experiments, a thin shell of epithelial cells located around the periphery of the tumour is created. OVCAR-3 cells are seen to possess more epithelial behaviours, expressing high amounts of the marker E-cadherin (green)^67^. While the majority of OVCAR-3 cells in the spheroid are epithelial, small clumps of mesenchymal cells are formed inside the tumour. This creates a polka dot effect in the observations of the neoplasm.

### Modelling framework

The model developed here is built upon a PhysiCell version 1.13.1 framework^68,69^. PhysiCell is an agent-based, multiscale, 3D framework used to perform in-silico simulations of biological processes. Cells move off-lattice along a continuous domain. Substrates are tracked on a 3D discrete mesh into which the domain is divided. A detailed overview of the cell and substrate mechanics can be found in the PhysiCell overview paper by Macklin et al.^68,69^.

A cell cycle model with four stages is included. Mitochondrial consumption of oxygen is found to increase during the G1/S transition^70^, suggesting a dependence on oxygen in the microenvironment for the rate at which a cell leaves the G1 stage of the cell cycle. Density-dependent cell-cell contacts have also been found to lead to arrest in the G1 phase of the cell cycle^71^. Therefore, variations in the cell cycle rate due to inter-cellular and intra-cellular conditions are incorporated into the G1 stage, with fixed rates of exiting stages S, G2, and M. Including the cell cycle as a four stage process rather than using an overall proliferation rate allows us to more accurately determine population dynamics by including realistic influences of the cellular conditions on the cycling rate. While EMT does appear more likely to occur in the G1 phase, cells are not limited to transitioning only during this stage of the cell cycle. EMT induced by TGF-*β*1 was found in cells synchronized at the G1/S phase but not in those synchronized at the G2/M phase^72^. However, despite increased blebbing during the M phase of cell division leading to a reduction of EMT-like phenotype, the transition can still be completed during mitosis^73^. We therefore, for simplicity, assume that the probability a cell undergoes EMT is independent of the stage of the cycle the cell is in.

1 $$r={b}_{c}\cdot {c}_{c}\cdot {p}_{c}\cdot {o}_{c}\cdot \left(1-\frac{n}{K}\right).$$

To model the cell cycle, we denote *b*_*c*_ as the base cycle rate, *c*_*c*_ as the cadherin impact on the cycling rate, *p*_*c*_ as the pressure impact on the cycling rate, and *o*_*c*_ as the oxygen impact on the cycling rate. These parameters are described in Section “Cell Cycle” and are used to calculate the rate at which a cell leaves the G1 stage of the cell cycle using Equation (1), where *r* is the cycling rate, *n* is the current population, and *K* is the carrying capacity. The carrying capacity is set to 6500 to allow a maximum population similar to that observed in the experimental data. Due to the cycling rate variability being enforced in the G1 stage of the cycle, there is a time delay in the effect of this rate and the tumour can reach populations higher than that specified in the carrying capacity of the logistic growth term. We include the logistic growth term with a carrying capacity to reduce the growth rate of large tumours, observed experimentally in which populations plateau.

Assuming on average that cells leave G1 with a rate of 1/11 h^−1^^74^, these parameters allow a maximum cycling rate of 8/11 h^−1^ to leave G1. The length of the cell cycle would therefore vary between around 14 h in the optimal conditions for cell proliferation and 24 h in the poorest. The remaining steps in the cell cycle (S, G2, and M) are unaffected by the microenvironmental conditions in the model.

**Fig. 2:**
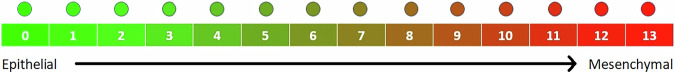
Appearance of cells during the simulations based on their current N-cadherin rating. Cells can take fourteen different values, with ratings 0-6 classified as epithelial and 7-13 classified as mesenchymal. There is an average rating of three for epithelial cells and ten for mesenchymal cells. The colour of each cell during the simulations represents where along this scale they are placed.

In the modelling framework, cells are assigned a rating along a scale of their epithelial to mesenchymal phenotype running from zero (epithelial) to thirteen (mesenchymal), as illustrated in Fig. 2. The maximum rating is set to thirteen to allow an equally weighted assignment of epithelial cells to ratings of 0-6 and mesenchymal to 7–13. Moreover, an average rating value for epithelial (rating 3) and mesenchymal (rating 10) cells can be set as the default if required. On each iteration, a cell has a probability of increasing N-cadherin rating depending on various inter-cellular and intra-cellular conditions such as hypoxia and current cell rating, discussed in more detail in Sections “Cadherin Rating” and “Jump Probability—Incorporation of EMT”. Only a maximum of one step can be made on each iteration, meaning jumps of multiples steps at once are not possible.

The EMT impact parameters incorporated into Equation (2) are discussed in Section “Jump Probability—Incorporation of EMT” for OVCAR-3 and SKOV-3 cells, showing their cumulative effect. This jump probability, *p*, determines if a cell will increase its N-cadherin rating during each 6 min iteration of the computer simulation, leading to a slight increase in mesenchymal-like behaviours highlighted in Section “Cadherin Rating”. Six minutes is chosen as the iteration length to ensure simulations remain relatively fast while keeping the timesteps as minimal as possible to increase simulation precision.

2 $$p={c}_{e}+{o}_{e}+{s}_{e}-{c}_{e}\cdot {o}_{e}-{c}_{e}\cdot {s}_{e}-{o}_{e}\cdot {s}_{e}+{c}_{e}\cdot {o}_{e}\cdot {s}_{e}.$$

**Fig. 3:**
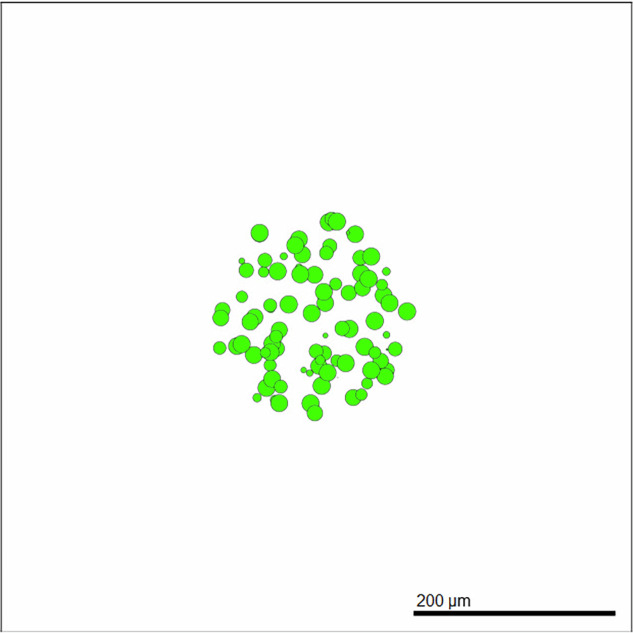
Cross-section of an example tumour at time *t* = 0. 1027 cells are randomly placed within a sphere of radius 120 microns around the centre of the domain. Snapshots such as this show a *z* = 0 cross-section of the spheroid at the respective time of the simulation, with the colour of the cells showing the current N-cadherin rating ranging between green (epithelial) to red (mesenchymal).

In Equation (2), *c*_*e*_, *o*_*e*_, *s*_*e*_, denote the cell line specific, weighted cadherin EMT impact, oxygen EMT impact, and signal EMT impact parameters as discussed in more detail in Section “Jump Probability—Incorporation of EMT”. Since EMT is a stochastic process, we take a probabilistic approach to determine how likely a cell is to jump up the N-cadherin rating on each iteration of the simulation. Each *y* variable in Table 1 is viewed as the probability that an event occurs, the event in this case being the cell moving up the N-cadherin rating on an iteration as a result of the respective intra-cellular or inter-cellular condition. We assume for simplicity that each of these three variables assigned to each cell is independent of each other. The total probability that a cell jumps up the N-cadherin rating on an iteration is therefore the probability that any of these events occur, i.e., the probability of their union. This probability is given in Equation (2). The parameters in Equation (2) are cell line dependent, the details of which are highlighted in Sections “OVCAR-3: Impact of microenvironment” and “SKOV-3: Impact of microenvironment”. When a parent cell divides, the N-cadherin rating in the model is conserved to the daughter cells. An example of the initialization is given in Fig. 3. This depicts a cross-section of an OVCAR-3 tumour through the *z* = 0 plane at time *t* = 0 in which all cells are epithelial with a N-cadherin rating of zero.

**Table 1:** Hill function parameters used for the variables in the EMT probability equation

EMT probability related parameters					
*y*	*x*	*base*	*sat*	*K*_50_	*p*
Cadherin EMT Impact (${c}_{e}^{* }$)	N-cadherin Rating	0	1	6.5	4
Oxygen EMT Impact (${o}_{e}^{* }$)	Oxygen Concentration	1	0	1	4
Signal EMT Impact (${s}_{e}^{* }$)	Signal Concentration	0	1	3	4

The table shows how the N-cadherin rating, oxygen concentration, and signal concentration that a cell is in can influence the EMT probability of a cell. Hill functions are used to quantify the impact of these conditions on the cell EMT probability according to the parameters shown in the table. The details can be found in Section “Cadherin rating”.

Here, we initialize the simulations with 1027 cells in a 3D spheroid scattered randomly within a sphere of radius 120 microns. This is to recreate the initial conditions as closely as possible to those used experimentally, in which repeats using spheroids with a mean of 1027 cells were placed. Both oxygen and bystander signal substrates are absent at initialization. Oxygen has a constant influx into the system through Dirichlet boundary conditions applied on all boundaries of the domain set to 38 units. This is because the in-vitro tumours can only access oxygen through the tumour edges rather than any internal supplies such as vessels. The signal substrate is given Neumann boundary conditions with no constant influx. The model is simulated for 96 h in which all cells are initialized with an equal N-cadherin rating, specified for each simulation.

To investigate EMT, cells are initiated with a N-cadherin rating of zero. It is assumed that only EMT can occur since we take the tumour to be in its primary location^75^. MET, the reverse process, primarily only occurs when the mesenchymal cells have relocated and stabilized in a new environment^76,77^. This MET process will be discussed in more detail in Sections “Impact of MET and Initial Conditions” and “Modelling Mesenchymal to Epithelial Transition”.

### OVCAR-3 Results

**Fig. 4:**
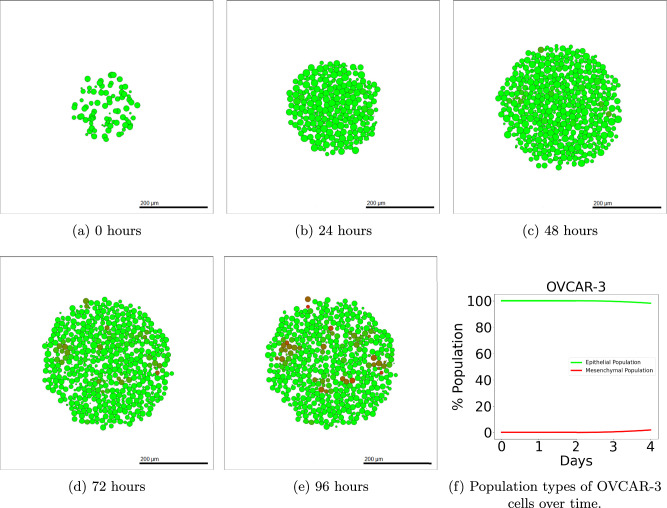
Simulated OVCAR-3 tumour at different time points using default parameter values. The initial placement of OVCAR-3 cells for the simulation is shown in (**a**). Cells after one day (**b**) and two days (**c**) show that initially, very small amounts of EMT occur in the first 48 h. Due to the bystander effect, clumps appear rapidly towards the latter simulation times, as shown after three days (**d**) and four days (**e**), in which small red mesenchymal clumps begin to form. Population types are shown in (**f**), with epithelial (green) and mesenchymal (red) tumour proportions over time with 95% confidence intervals.

Details of the methods used to simulate OVCAR-3 tumours can be found in Section “OVCAR-3: Impact of microenvironment”. After simulation completion, we can investigate how the tumours change in both composition and size over time. Figure 4 shows simulated cross-sections of an OVCAR-3 tumour after (a) 0 h, (b) 24 h, (c) 48 h, (d) 72 h, and (e) 96 h. After one day (b) there is very little change in the tumour composition as cells remain almost purely green, suggesting no change in their N-cadherin ratings from zero. After two days (c) small, faintly mesenchymal clumps form on the left and right of the tumour. Upon completing day three (d), early stages of clump formation begin to appear and multiple patches of red mesenchymal cells show scattering throughout the tumour. After the final day (e), multiple clumps of mesenchymal cells have advanced throughout the N-cadherin rating and progressed rapidly through the stages of EMT, showing close agreement to the observation seen in Fig. 1 (a).

We observe that despite the small red clumps, the majority of cells remain epithelial with a low N-cadherin rating. While the volume of these clumps appears negligible in comparison to the volume of the tumour, these clusters of mesenchymal cells cannot be overlooked. Upon breaking away, these cells can relocate and have a key responsibility in the metastasis of the tumour^78^. These clusters can perform collective migration throughout the body despite the lack of individual cell adhesion^79,80^. The exact procedure by which this is carried out and made viable is relatively undocumented.

**Fig. 5:**
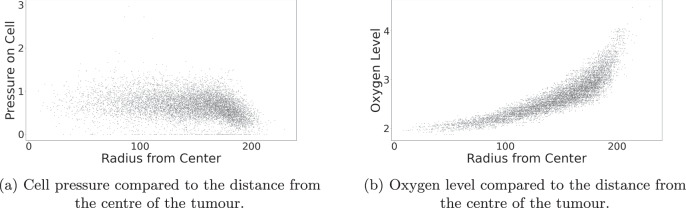
Conditions of cells with respect to the position in the tumour. Each black dot in the subfigures represents a cell at the final simulation time point. **a** shows how the trends in the dimensionless pressure on the cells are affected by the distance of the cell from the centre of the tumour. Using a trial simulation initialized with a tumour of OVCAR-3 cells, results show a general negative trend between the radius from the centre of the tumour and the pressure on a cell. Pressure is generally lowest for cells at high radii, suggesting those on the tumour surface are under lower pressure from the neighbouring cells. **b** shows a clear positive trend between surrounding oxygen levels for a cell and the radius of the cell from the centre of the tumour, confirming those cells on the surface of the tumour have access to more oxygen than those in the interior.

Moreover, each cell in the model has a pressure exerted on it by neighbouring cells, changing the cell cycling rate. We can compare the pressure each cell is under to its proximity to the centre of the tumour taken as the origin of the domain, shown in Fig. 5 (a). Qualitatively speaking, there is a general negative correlation between the distance a cell is from the centre of the tumour and the pressure acting upon it. This is in agreement with the results found in the literature^81^, allowing us to implement pressure-dependent behaviours to the cells in future model adaptations with confidence. Figure 5 (b) shows the oxygen levels throughout the tumour for each cell. Hypoxia is shown to be achieved in central areas of the tumour in which oxygen levels are lower than observed around the tumour exterior.

### SKOV-3 Results

**Fig. 6:**
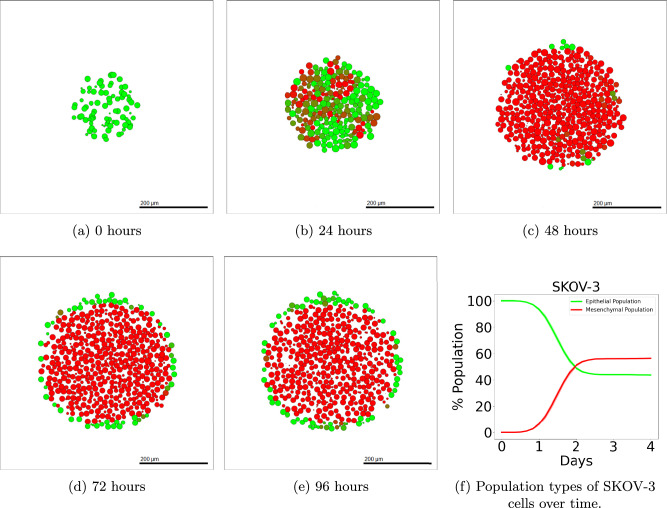
Simulated SKOV-3 tumour at different time points using default parameter values. The initial placement of SKOV-3 cells for the simulation is shown in (**a**). After only one day (**b**), high levels of EMT have already occurred throughout the tumour, with completed EMT observed in almost all interior cells after two days (**c**). A red mesenchymal pool forms inside the tumour after three days (**d**) and four days (**e**) with a green epithelial layer created around the periphery. Population types are shown in (**f**), with epithelial (green) and mesenchymal (red) tumour proportions over time with 95% confidence intervals.

Details of the methods used to simulate SKOV-3 tumours can be found in Section “SKOV-3: Impact of microenvironment”. SKOV-3 tumour simulations give drastically contrasting results to that seen in OVCAR-3 spheroids. Due to the increased jump probability, cells progress through the N-cadherin ratings faster. Figure 6 shows simulated cross-sections of a SKOV-3 tumour after (a) 0 h, (b) 24 h, (c) 48 h, (d) 72 h, and (e) 96 h. Large areas of the cross-section of the tumour begin to rapidly undergo EMT, creating a blend of epithelial and mesenchymal cells in the neoplasm as seen after one day in Fig. 6 (b). After 48 h of simulated time (c), a clear majority of interior cells have fully undergone EMT and have a N-cadherin rating of around thirteen. Occasional green epithelial cells arise around the tumour periphery where oxygen has increased above the threshold value. After 72 and 96 h in (d) and (e) respectively, the epithelial shell begins to form and creates a solid coating around the primarily mesenchymal tumour. The SKOV-3 tumours show a close agreement to experimental observations shown in Fig. 1 (b). The outer shell of epithelial cells remains thin, with only mesenchymal cells making up the interior of the tumour.

### Model analysis: comparison with experimental data

**Fig. 7:**
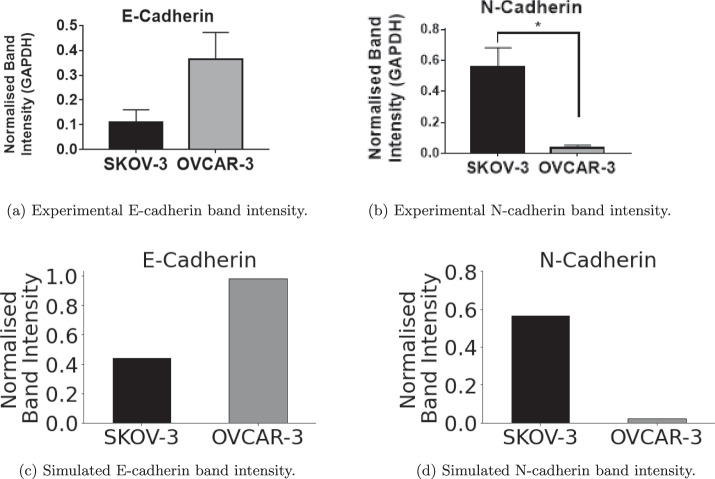
Band intensity of E-cadherin and N-cadherin for the in-vitro and in-silico experiments. Results after 96 h from the in-vitro biological experiments show the normalized band intensities of E-cadherin (**a**), a marker for epithelial cells, and N-cadherin (**b**), a marker for mesenchymal cells. These are compared with in-silico results taken after 96 h of simulated time. Proportions of cells classified as epithelial (E-cadherin) and mesenchymal (N-cadherin) for both cell lines are recorded, shown in (**c**) and (**d**) respectively.

Figure 7 shows the proportion of epithelial (E-cadherin) and mesenchymal (N-cadherin) cells in the final population of the tumour. Figure 7 (a) and (b) show the in-vitro results while (c) and (d) show the results found in-silico using the mathematical model. 95% Confidence interval bars are present in Fig. 7 (c) and (d). However, due to the consistency across results from ten simulation repetitions, the intervals are not visible in the bar charts. OVCAR-3 cells finish with a vast majority of epithelial cells in both the experimental and computer simulation results. SKOV-3 cells have a majority of mesenchymal cells, however, due to the outer shell being solely epithelial, SKOV-3 tumours have much higher proportions of epithelial cells than OVCAR-3 has mesenchymal. The simulation outputs are in qualitative agreement with the experimental results, with E-cadherin expression around three times higher in OVCAR-3 tumours than in SKOV-3 tumours. N-cadherin expression is negligible in OVCAR-3 tumours when compared to expressions in SKOV-3 tumours.

### Sensitivity analysis

Sensitivity analysis is a crucial part of any mathematical model, quantifying the sensitivity of each parameter on the output of a simulation. By varying these input parameters, we can calculate the variation in the output. Multiple aspects of the output can be investigated, including information on the cell populations or the substrate concentrations. We use a form of Latin Hypercube Sampling to perform global sensitivity analysis. The details of the methods used are described in the Supplementary Material (SM). Using the Pearson Product Correlation (*PCC*) value for different parameters tells us those which have the highest impact on the model output. We compare both how the size of the tumour and the composition of the tumour changes with fluctuations in the parameters used in Eqs. (1) and (2) for the two cell lines investigated. Generally, most parameters incorporated into these equations have a notable impact on at least one form of output. Parameters used in Eq. (1) unsurprisingly tend to have more impact on the final simulated tumour size than the mesenchymal fraction for both OVCAR-3 and SKOV-3 tumours. This is due to changes in the cycling rate having a direct link to the proliferation observed during the simulation. Parameters used in Eq. (2) have less impact on the final OVCAR-3 tumour populations but a much larger impact on the tumour composition. Increasing the parameters in Eq. (2) increases the probability of cells undergoing EMT. This impact is more observable in OVCAR-3 tumours where the mesenchymal clumps can be formed throughout the tumour rather than only in the interior. SKOV-3 spheroids generally reach complete EMT in the tumour regardless of small changes in the parameter values used in Eq. (2), as shown in Supplementary Fig. 5. As a result, minor perturbations in the EMT impact weightings used for SKOV-3 have little impact on whether complete interior EMT is achieved.

### Impact of MET and initial conditions

It has been observed that mesenchymal cells which have undergone EMT and have relocated to a secondary location may undergo MET at this new location^82^. This allows the cell to re-obtain the epithelial phenotype and behaviours previously exhibited to encourage stability and enhanced proliferative abilities^77^. MET has been observed in OVCAR-3 cell lines in which partial EMT has been completed^83^. SKOV-3 cells also show capabilities of showing molecular changes consistent with MET, transitioning from elongated to cuboidal shapes^84^. Here, we explore the effects of MET by including a probability in which cells can jump down in N-cadherin rating, *q*. Cells continue to traverse through the N-cadherin ratings, jumping by only one step at a time. However, in this section, they are able to move either up or down during each iteration to allow for this MET process to be captured by the model.

**Fig. 8:**
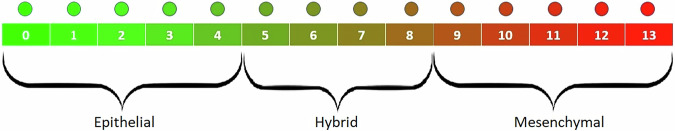
Classification of N-cadherin ratings when hybrid cells are included in the model. When including MET, a hybrid state is introduced into the classification of cells. Cells rated 0-4 are now classified as epithelial, 5-8 as hybrid, and 9-13 as mesenchymal. This ternary classification allows for more detailed results and easier comparisons with past models as shown in Sections “OVCAR-3 with MET” and “SKOV-3 with MET”.

To investigate the effects of heterogeneous cellular composition, a “hybrid” cell classification is now included into the model. Instead of only including epithelial and mesenchymal cells, the population is divided into three subgroups. These subgroups now include a hybrid state for the cells, giving us more precise results when tracking population types over time by providing extra categories for classification. Cells with a rating of four or less are now classified as epithelial, five to eight inclusive as hybrid cells, and nine or more as mesenchymal (See Fig. 8). The tumour is initialized with each cell type separately to investigate the temporal impact of the starting tumour population type. This could ultimately provide an overview of how treatments for patients with tumours composed of varying cell types are likely to differ with time. Epithelial cells are initialized with a rating of zero, hybrid with a rating of seven, and mesenchymal with a rating of thirteen.

As described in Section “Modelling Mesenchymal to Epithelial Transition”, we can calculate the probability for a cell to jump down in rating during each iteration using Equation (3). Cells are assigned a higher probability when their N-cadherin rating is lower and the cell is in higher concentrations of oxygen. Since no evidence was found in the literature of any bystander signal impacting MET rates, the concentration of the bystander signal was not incorporated into this equation.

3 $$q={c}_{m}+{o}_{m}-{c}_{m}\cdot {o}_{m}.$$

#### OVCAR-3 with MET

##### Epithelial

**Fig. 9:**
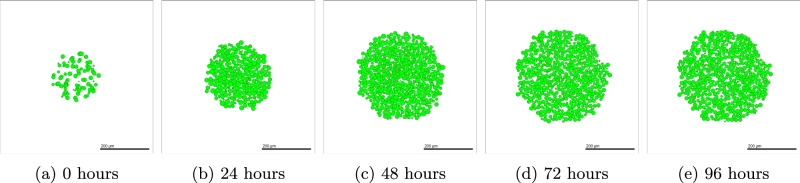
OVCAR-3 tumour over four days of simulated time, initialized with epithelial cells. The initial placement of OVCAR-3 cells with an N-cadherin rating of zero is shown in (**a**). Minimal EMT occurs in the first day (**b**), with only very faint areas of darker green cells appearing after two days (**c**), suggesting very little EMT has occurred at this point. The amount of EMT undergone after three days (**d**) and four days (**e**) remains negligible, with all cells remaining purely epithelial over time.

OVCAR-3 tumours initialized with epithelial cells no longer obtain the defined isolated clumps of mesenchymal cells observed in Fig. 4 during the simulation. Instead, the MET probabilities are too high to allow any notable amount of EMT to occur, as shown in Fig. 9. The tumour size grows rapidly over time since the composition of the tumour remains mostly epithelial. Epithelial cells have the fastest cycling rate and so the tumour can proliferate at a faster rate than those initialized with hybrid or mesenchymal cells.

Due to the lack of mesenchymal cells, even after 96 h of simulated time, the adhesion between cells in the tumour remains high. The cells remain densely packed and part of the main tumour rather than breaking away and losing contact with other cells. This suggests that metastasis would be unlikely to occur at this point since the tumour is one rigid structure.

##### Hybrid

**Fig. 10:**
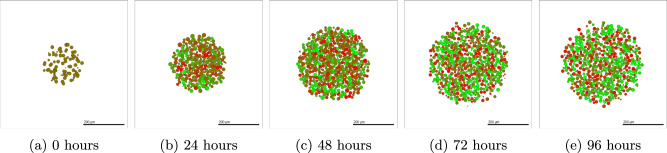
OVCAR-3 tumour over four days of simulated time, initialized with hybrid cells. The initial placement of OVCAR-3 cells with an N-cadherin rating of seven is shown in (**a**). After one day (**b**), the tumour is made of a mixture of epithelial, hybrid, and mesenchymal cells, each scattered what appears to be at random throughout the tumour, with similar observations seen after two days (**c**). After three days (**d**), small collections of epithelial cells can be observed in among the scattering of individual epithelial, hybrid, and mesenchymal cells, seen further after four days (**e**).

OVCAR-3 tumours initialized with hybrid cells result in a complete mix of epithelial, hybrid, and mesenchymal cells. No ordering or formation of clumps is visible despite the bystander effect present, as shown in Fig. 10. Population sizes of the cell types appear generally similar with no clear majority of epithelial, hybrid, and mesenchymal cells present.

The tumour initialized with hybrid cells shows very high plasticity after only 24 h of simulated time, as seen in Fig. 10 (b). Following one day of simulated time there is a large population of green and red cells at the extremities of the epithelial-mesenchymal scale. This is unlike other initial conditions in which immediate changes in the tumour appearance are not observed as clearly. This suggests that hybrid cells in the model have the lowest stability and can fluctuate between states more readily than the more stable epithelial or mesenchymal cell type. Partial EMT or hybrid E/M states are common in ovarian cancer and allow cells to retain both epithelial and mesenchymal characteristics. These cells often exhibit continued proliferation while gaining motility and resistance to therapy, contributing to metastasis^85^.

##### Mesenchymal

**Fig. 11:**
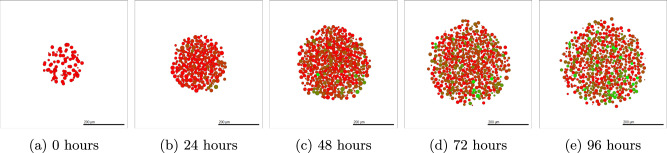
OVCAR-3 tumour over four days of simulated time, initialized with mesenchymal cells. The initial placement of OVCAR-3 cells with an N-cadherin rating of thirteen is shown in (**a**). Minimal MET occurs in the first day (**b**), with areas of green epithelial cells appearing after two days (**c**). This patch of epithelial cells in the bottom right section of the tumour continues to grow after three days (**d**) with areas of epithelial cells observed throughout the tumour after four days (**e**), despite the vast majority of cells remaining mesenchymal during the simulation.

When OVCAR-3 tumours are initialized with mesenchymal cells, the majority of the cells remain mesenchymal throughout the 4 day simulation. After the final simulation output, a scattering of epithelial cells is present throughout the tumour with no immediate observable patterning, as shown in Fig. 11.

The OVCAR-3 tumour remains mostly mesenchymal over time, with a small number of epithelial cells appearing in the final tumour in Fig. 11 (d) and (e). A number of cells can be seen escaping the main tumour clump and moving freely out into the domain. The tumour appears less grouped together with more empty space between the cells than seen in Figs. 9 and 10. This is due to the high mesenchymal population decreasing the adhesion strengths within the tumour and the extra cell motility creating a less rigid tumour structure. This model resembles biologically the mesenchymal tumour cells with enhanced migratory capacity, favouring tumour cell dissemination over proliferation.

##### Temporal Dynamics

**Fig. 12:**
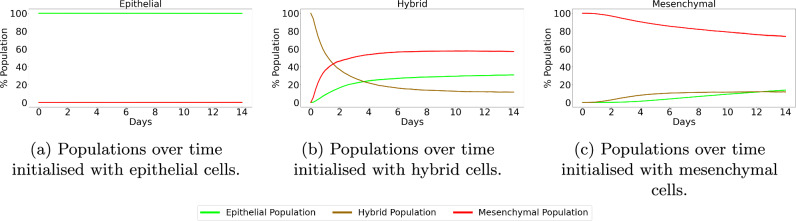
Epithelial vs hybrid vs mesenchymal OVCAR-3 cell populations found in-silico including MET. SKOV-3 tumours are initialized with epithelial (**a**), hybrid (**b**), or mesenchymal (**c**) cells. Simulations are run for 96 h with the cellular proportions of the OVCAR-3 tumour composition recorded every hour. Curves show the populations of epithelial (green), hybrid (brown), and mesenchymal (red) cells during the simulation. Confidence intervals of 95% are present in each plot, taken from ten repeats of the simulation. However, due to the lack of substantial stochastically in the model, these intervals are not visible in the plots.

Here, we study the temporal evolution of cell populations to understand the dynamics and cellular transitions as shown in Fig. 12. This is compared with experimental data obtained by Ruscetti et al.^86^ for prostate cancers, obtained using the PKV cell line. The experiments by Ruscetti et al.^86^ show the epithelial-mesenchymal plasticity in the PKV cell line cultured in vitro. Population fractions of each phenotype are tracked over the course of 14 days, obtaining the temporal dynamics of the tumour. The PKV cells were isolated using fluorescence-activated cell sorting, identifying epithelial, hybrid, and mesenchymal-like cells during the in-vitro experiment. Cells undergoing EMT express green fluorescent protein, suggesting higher rates of transition to a mesenchymal phenotype.

The comparisons between SKOV-3 and OVCAR-3 tumours in previous sections highlight the necessity for identifying potential differences in cell line characteristics. Despite this, different cell lines may still possess similar general trends and provide tentative insights into the predicted behaviour of others for model validation. The qualitative trends between the PKV cell line experimental results found in vitro by Ruscetti et al.^86^ and the OVCAR-3 cell line model results found here in-silico are in close agreement. To remain consistent with the results found in-vitro, we extend the time of the simulation from four days to fourteen. Simulations initialized with epithelial cells remain almost entirely epithelial throughout the simulation, with only small fluctuations in the N-cadherin rating of cells, as shown in Fig. 12 (a). In Fig. 12 (b), hybrid cells can go either way along the cadherin scale, with a notable population of epithelial, hybrid, and mesenchymal cells all present at the final time. Simulations initialized with mesenchymal cells remain mostly mesenchymal, with a small population of both epithelial and hybrid cells forming over time.

**Fig. 13:**
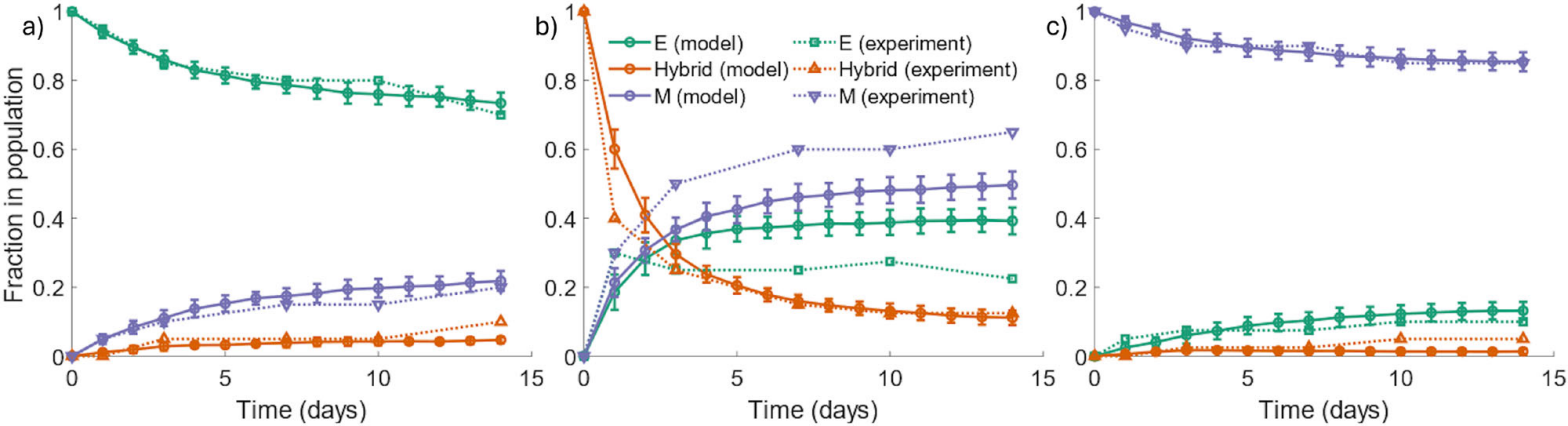
Epithelial vs hybrid vs mesenchymal cells populations found by Tripathi et al.^87^. Each panel shows the cell population of the cell types over time for both an in-vitro experiment performed by Ruscetti et al.^86^ (dotted curves) and in-silico simulation results of a model designed by Tripathi et al.^87^ (solid curves). Epithelial (E), hybrid, and mesenchymal (M) cell populations are shown using green, orange, and purple curves respectively. Cells initialized with either epithelial (**a**) or mesenchymal (**c**) cells generally remain with the respective cell type as the majority of the tumour at the final time of the simulation. Tumours initialized with a hybrid population (**b**) show lower stability, with cells rapidly transforming into either mesenchymal or epithelial cells.

Figures 12 and 13 confirm the importance of initial conditions on the tumour development, with (a) and (c) showing the stability in tumours initialized with epithelial or mesenchymal cells. While small changes occur in the tumour composition, the majority of cells in these scenarios remain the same type as those they were initialized with. A tumour initialized with hybrid cells (b) shows increased plasticity and fluctuation in the cell types, with mesenchymal and epithelial cells both making up a large proportion of the overall tumour population.

#### SKOV-3 with MET

**Fig. 14:**
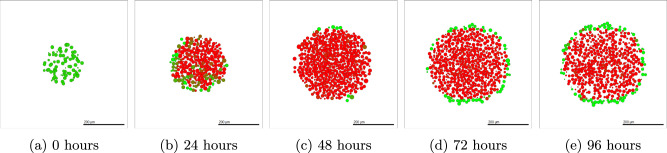
SKOV-3 tumour over four days of simulated time, initialized with epithelial cells. Epithelial SKOV-3 cells are placed in the domain with an N-cadherin rating of zero (**a**). After one day, the majority of the tumour has undergone full EMT (**b**), showing a large area of red mesenchymal cells within the tumour. All interior cells complete EMT within two days (**c**), with occasional cells around the periphery converting back to epithelial cells as a result of high oxygen levels. This epithelial shell becomes more prominent after three days (**d**) and four days (**e**), where the outer layer of epithelial cells surrounds the interior pool of mesenchymal cells.

We can perform a similar experiment using SKOV-3 tumours. Since SKOV-3 cells are assumed to progress through EMT more rapidly than OVCAR-3 cells, the tumour progresses to the steady state of mesenchymal cells in the interior with a shell of epithelial cells around the exterior very rapidly. This leads to less dependence on the initial condition of the tumour cell type. Figure 14 shows the progression of the tumour over four days when initialized with epithelial cells. After 24 h of the simulation, the majority of the cells become mesenchymal and have undergone EMT, as shown in Fig. 14 (b). Few epithelial cells remain due to sufficient oxygen levels preventing EMT from occurring, with the vast majority of interior cells undergoing complete EMT within the first day. Midway through the simulation, as shown in Fig. 14 (c), all cells other than those initiating the formation of the epithelial cells along the periphery undergo complete EMT. These interior cells remain mesenchymal for the remainder of the simulation.

**Fig. 15:**
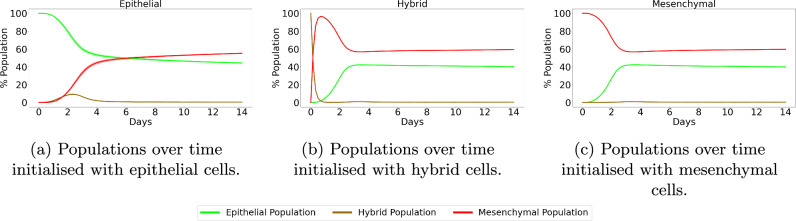
Epithelial vs hybrid vs mesenchymal SKOV-3 cell populations found in-silico including MET. SKOV-3 tumours are initialized with epithelial (**a**), hybrid (**b**), or mesenchymal (**c**) cells. Simulations are run for fourteen days with the cellular proportions of the SKOV-3 tumour composition recorded every hour. Curves show the populations of epithelial (green), hybrid (brown), and mesenchymal (red) cells during the simulation. Confidence intervals of 95% are present in each plot, taken from ten repeats of the simulation. However, due to the lack of substantial stochastically in the model, these intervals are not visible in the plots.

Figure 15 shows the populations of each cell type over time. In all initial conditions, mesenchymal cells rapidly become the main cell type in the SKOV-3 tumour. The epithelial cell population fraction gradually increases in Fig. 15 (b) and (c). This is due to the tumour periphery expanding outwards and becoming more oxygenated due to the Dirichlet conditions from the domain boundary. This increased oxygen allows the threshold to be reached deeper into the tumour and the thickness of the epithelial shell to be increased. Cells mostly remain divided into either mesenchymal or epithelial, with very few hybrid cells appearing throughout the tumour. This agrees with the biological observations in the experiments shown in Fig. 1 (b), where there is a clear division between the red pool of mesenchymal cells in the tumour core and green epithelial cells around the edge. Regardless of the initial conditions in the model, final population sizes for each type of SKOV-3 cell remain relatively consistent due to the fast rate at which tumour stability is reached compared to OVCAR-3 tumours.

## Discussion

The role of EMT has been shown to have a key impact on OVCAR-3 and SKOV-3 tumours over time. While on the surface it may appear EMT is a binary process in which a switch is simply turned on, the complex dynamics in the background lead to a microenvironment-dependent, heterogeneous tumour layout. Few models created previously have investigated this continuous perspective of EMT with such a spatially dependent, heterogeneous approach. OVCAR-3 and SKOV-3 cells use similar rules in the mathematical model, with identical Hill functions used in the generation of parameters required for the EMT and cycling rate equations. By varying only the factor at which these parameters are larger in SKOV-3 cells than OVCAR-3 cells and incorporating an oxygen threshold in which SKOV-3 cells become epithelial, the biological observations seen in-vitro can be accurately recreated. These changes are sufficient to induce the drastic differences seen between the cell lines experimentally.

The bystander effect is proven to generate results similar to those found experimentally. By using biological observations for calibration, we test our model against other data sets, observed in Fig. 13. The results show that the developed mathematical model can qualitatively predict the biological observations, indicating the usefulness of the model in exploring processes involved in EMT, MET and potentially to study responses to the therapy.

Sensitivity analysis on the model demonstrates that the outputs of interest are sensitive to most parameters involved in generating the rate at which a cell cycles (Equation (1)) and undergoes EMT (Equation (2)). SKOV-3 tumours have the unexpected behaviour of the mesenchymal fraction appearing independent of the parameter values used in the jump probability (Equation (2)). Instead, the fraction is more dependent on the parameters used in the cell cycling rate in Equation (1). This is due to the fact that oxygen has Dirichlet conditions on the boundary edges, meaning cells able to get closer to the edges are in higher concentrations of oxygen. Higher cycling rates allow the tumour to expand and reach the boundary edges faster, allowing oxygen to reach further into the tumour surface and creating a thicker shell of epithelial cells around the tumour periphery.

The inclusion of MET in Section “Modelling Mesenchymal to Epithelial Transition” highlights the importance of including all relevant processes on the cells studied. Including MET restricts the ability of the chemical signal to create mesenchymal clumps in OVCAR-3 tumours via the bystander effect. The tumours without MET ability are more representative of pre-metastatic tumours rather than those which have relocated into a secondary location. The detachable mesenchymal clumps and lack of clear overall structure to the tumour generally make OVCAR-3 tumours more harmful than SKOV-3 tumours. When including the process of MET alongside EMT, differences in the final tumour layouts appear. The clumps seen in OVCAR-3 tumours are less apparent in the neoplasm, while SKOV-3 tumours remain possessing a similar green epithelial shell around the pool of central red mesenchymal cells. By changing initial conditions, we can see the importance of how we set the tumour composition used when starting a simulation. The bystander effect induces a chain reaction, encouraging cells in the proximity of surrounding mesenchymal cells to undergo EMT at an increased rate and become mesenchymal themselves. In SKOV-3 tumours the jump probability is large enough to reach stability within four days as EMT can be achieved in a shorter period of time than in OVCAR-3 tumours. This stability is seen in biological observations in which the interior of the tumour completes EMT and is made exclusively of mesenchymal cells, while the exterior cells remain epithelial with time upon reaching a certain proximity of the domain boundaries. These findings highlight the need for accurate diagnosis when a patient is seen with ovarian cancer, in both the tumour size and composition. We see major differences between the simulations involving only EMT and those also including MET.

The developed multiscale model shows a reasonable level of quantitative and qualitative agreement with experimental data^86^ and previous model results^87^. Despite both different methods of fluorescence analysis performed between the varying cell lines, the general trends found between Ruscetti et al.^86^ and the mathematical model described above show similar qualitative results. While Ruscetti et al.^86^ use a different cancer type (prostate) to that used for our model, this agreement allows us to have a certain level of confidence when extrapolating the model and parameter values beyond those in scenarios investigated experimentally. Doing this creates an opportunity to explore heterogeneous tumour cells with intra-tumour and inter-tumour variabilities such as a specific size, cell line, and mesenchymal composition. This potentially allows one to develop a digital twin and test different treatment dosages and administrative intervals to find the best outcome for each specific patient. Without the aid of mathematical models, creating digital twins in a laboratory setting and investigating multiple different conditions is virtually impossible. These digital twins can conclude which treatment protocol is optimal, thus improving ovarian cancer survival rates and reducing the number of deaths it causes each year.

## Methods

Concentrations are calculated using a partial differential equation (PDE) developed in BioFVM^68^. The substrate domain is split into a discrete mesh comprised of small cubes referred to as voxels. Neighbouring voxels allow substrates to diffuse across the domain at rates dependent on substrate-specific diffusion coefficient values selected by the user. Decay terms are also included in the PDE, as well as any specific source and uptake rates as a result of processes such as cellular consumption or excretion. Equation (4) provides details of the PDE used to compute the substrate concentrations in each voxel, with each term described in Table 2. Substrate-related parameters can be found in Supplementary Table 3 in the Supplementary Materials.

4 $$\begin{array}{l}\frac{\partial \rho }{\partial t}=D{\nabla }^{2}\rho -\lambda \rho +S({\rho }^{* }-\rho )-U\rho \\\qquad+\,\sum _{{\rm{cells}}\,k}\delta ({\bf{x}}-{{\bf{x}}}_{k}){W}_{k}[{{\bf{S}}}_{k}({{\boldsymbol{\rho }}}_{k}^{* }-\rho )-{{\bf{U}}}_{k}\rho ]\,\text{in}\,\Omega\end{array}$$

**Table 2:** The list of parameters used in the PDE for substrate dynamics

Symbol	Meaning	Dimension
*ρ*	substrate density/concentration	substance/volume
*D*	diffusion coefficient	length^2^/time
*λ*	decay rate	1/time
*W*_*k*_	volume of cell *k*	volume
*x*_*k*_	position of cell *k*	length
*S*_*k*_	secretion rate of cell *k*	1/time
*ρ*_k_^*^	secretion saturation of cell *k*	substance/volume
*U*_*k*_	uptake rate of cell *k*	1/time

The table shows the biological meaning of each variable and parameter along with the corresponding units. Values for each parameter are substrate-specific.

### Cell cycle

**Fig. 16:**
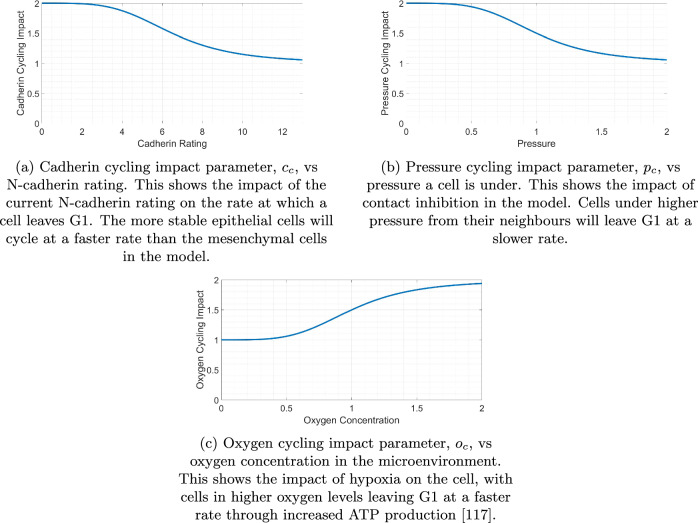
Dependence of cell cycling parameter values on cell conditions. Increased N-cadherin rating (**a**) and pressure a cell is under from neighbouring cells (**b**) decrease the cell cycling rate, while increased oxygen concentration (**c**) increases the cell cycling rate. Hill functions range between a maximum of two to a minimum of one.

Cells in the model progress through the cell cycle at varying rates depending on the conditions within both the cell and the microenvironment (see Fig. 16)^88^. Cells with a higher N-cadherin rating are assumed to have more mesenchymal phenotypic behaviours and so cycle slower than those possessing more epithelial characteristics^89^. We include this phenomenon by introducing a Hill function used in generating a cadherin cycling impact parameter, as shown in Fig. 16 (a). These Hill functions are given a Hill power of four. This value is small enough to allow for changes in the behaviours when minor adjustments are made in dependent variables, while being large enough to create non-linear correlations. This parameter is two for entirely epithelial cells, asymptotically approaching one for entirely mesenchymal cells. These Hill functions asymptotically approach their saturation value rather than reaching their exact value, meaning the value of this parameter will never be exactly one. The parameters that were used in generating these Hill functions are shown in Table 3. Cells under higher pressure due to combined repulsion forces from neighbouring cells also reduce the cell cycling rate, as shown in Fig. 16 (b)^90,91^. This is to encapsulate the effect of cells requiring empty space in the surrounding area to divide into^92^. We generate this pressure cycling impact parameter value using a similar Hill function to that seen in Fig. 16 (a), where the parameter value is two where no pressure is applied to the cell and tends to one where the pressure is two units. The unit of pressure is defined using a dimensionless analogue described in the documentation of Physicell^69^. With a greater concentration of oxygen available in the microenvironment, cells are able to increase adenosine triphosphate (ATP) production and therefore cycle faster^93–96^. This is incorporated into the model in Fig. 16 (c) by introducing an oxygen cycling impact parameter ranging from one in hypoxic conditions to two in oxygen-rich conditions. Due to the difficulty in quantifying the exact minimum and maximum parameter values in these functions, we assume that each cycling impact parameter fluctuates between one and two, meaning each variable has the capability of doubling the rate to exit G1. This assumption allows us to make qualitative conclusions regarding how the tumour size may depend on the intra-cellular and inter-cellular conditions.

**Table 3:** Hill function parameters used for the variables in the cycling rate equation

Cycling Rate Related Parameters					
*y*	*x*	*base*	*sat*	*K*_50_	*p*
Cadherin Cycling Impact (*c*_*c*_)	N-cadherin Rating	2	1	6.5	4
Oxygen Cycling Impact (*o*_*c*_)	Oxygen Concentration	1	2	1	4
Pressure Cycling Impact (*p*_*c*_)	Pressure	2	1	1	4

The table shows how the N-cadherin rating, oxygen concentration, and pressure that a cell is under can influence the cycling rate of a cell. Hill functions are used to quantify the impact of these conditions on the cell cycling rate according to the parameters shown in the table.

### Cadherin rating

The N-cadherin rating of a cell has a key influence on its phenotypic characteristics^97^. We use a number of Hill functions to build correlations between the current N-cadherin rating and the behaviour of a cell as given in Equation (5). Here, *y* denotes the response to a variable *x*, *base* and *sat* are the values that the Hill function can take for *x* = 0 and as *x* tends to infinity respectively, *p* is the Hill power to assign the steepness of the curve, and *K*_50_ is the half max, the value of *x* for which *y* is half way between the *base* and *sat* values.

5 $$y=\frac{(sat-base)\cdot {x}^{p}}{{K}_{50}^{p}+{x}^{p}}+base.$$

**Fig. 17:**
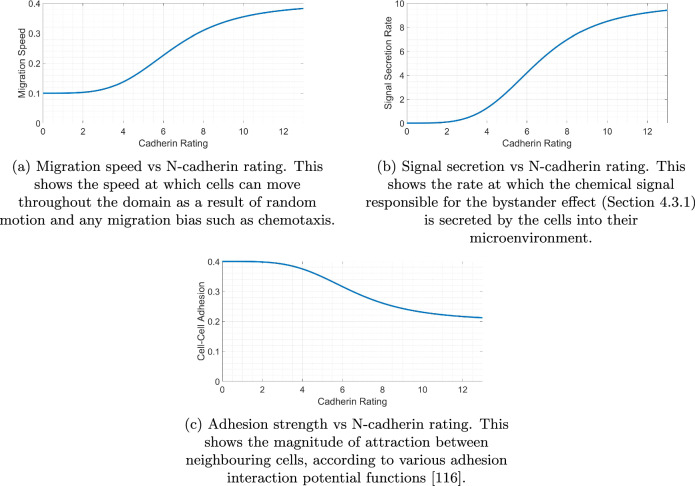
Dependence of different cell variables on the current N-cadherin rating of the cell. Increased N-cadherin ratings increase cell migration (**a**) and bystander signal secretion rate (**b**) while decreasing cell-cell adhesion strength (**c**).

Figure 17 shows the assumed quantitative trends with N-cadherin rating changes in different cell behaviours, such as migration speed (a), signal secretion rate (b), and cell-cell adhesion strength (c). Hill functions are used to generate these correlations, with relevant values shown in Table 4. Mesenchymal cells have been found to possess enhanced migratory tendencies^98,99^ and lower adhesion strength than seen in epithelial cells^100,101^. Here, the increased migration speed which is permitted for mesenchymal cells allows the cells to move with greater freedom through the domain and increases the probability of the cell leaving the tumour itself to reach the domain boundary. The decrease in adhesion strength allows the mesenchymal cells to break away from neighbouring cells and escape the tumour with greater ease.

**Table 4:** Hill function parameters used for the phenotypic behaviours

Cell Behaviour Related Parameters					
*y*	*x*	*base*	*sat*	*K*_50_	*p*
Migration Speed	N-cadherin Rating	0.1	0.4	6.5	4
Signal Secretion Rate	N-cadherin Rating	0	10	6.5	4
Cell-Cell Adhesion	N-cadherin Rating	0.4	0.2	6.5	4

The table shows how the N-cadherin rating can influence the behaviours of a cell in the model. Hill functions are used to correlate the rating to the migration speed, signal secretion rate, and cell-cell adhesion strengths.

In this context, bystander effects are the cumulative effects of various factors such as cytokines, growth factors, and exosomes secreted by cancer cells^102^. In the model, the bystander effect is responsible for the formation of the disjoint mesenchymal clumps observed in the OVCAR-3 hybrid tumour population, co-expressing epithelial and mesenchymal markers^103^. We assume that these bystander effects encourage epithelial cells in their proximity to undergo EMT. These factors secreted by mesenchymal cells help encourage changes in the cell phenotype and increase the rate at which cells undergo EMT^104,105^. This creates localized pockets of mesenchymal clusters in which high amounts of these secreted factors are present, further encouraging EMT in the surrounding cells. Such tumour heterogeneity is apparent in solid tumours where a mosaic expression of cadherins has been described in both primary and secondary epithelial ovarian cancer tumours^106,107^. Thus, we aim to capture these bystander effects and mechanisms of chemoresistance which are apparent in tumour spheroids by incorporating aspects of a localized tumour microenvironment including gradients of nutrient availability^108^. We assume that the signal secretion rate is increased with N-cadherin rating, meaning epithelial cells in the presence of mesenchymal cells are exposed to a higher concentration of signal and are more likely to increase in N-cadherin rating themselves, inducing the bystander effect.

### Jump probability—incorporation of EMT

Here, we assume that cells can have varying predicted rates of undergoing EMT. On each iteration of the simulation, a cell is given a probability of moving up through the N-cadherin rating and gaining more mesenchymal properties. We assume that the magnitude of this probability is dependent on factors in both the cell itself (the N-cadherin rating) and the microenvironment around it (the oxygen and bystander signal levels). These inter-cellular and intra-cellular conditions give rise to a stochastic process by which the N-cadherin rating of the cell is determined.

#### Impact of inter-cellular conditions

**Fig. 18:**
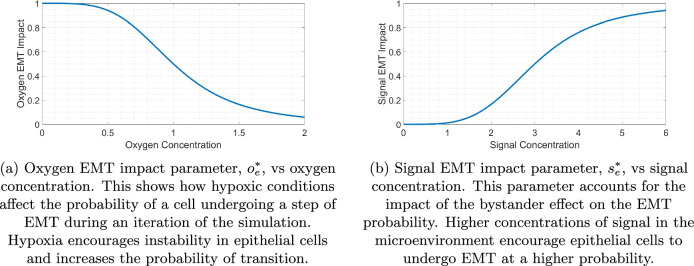
Dependence of the EMT rate parameters on the microenvironment. Increased oxygen concentration reduces the probability of EMT occurring in a cell (**a**) due to the lack of hypoxic conditions that encourage EMT. To create the bystander effect, an increased concentration of signal around a cell increases the rate of EMT (**b**). This ensures epithelial cells in the presence of signal-secreting mesenchymal cells are more likely to undergo EMT and form mesenchymal clumps within the tumour.

Following experimental observations, we hypothesize in our model that two main factors in the microenvironment contribute to EMT within epithelial cells. Hypoxic conditions have been found to encourage EMT in cancer cells by generating various signalling pathways and activating transforming growth factor TGF-*β*^109,110^. This is achieved in the model using a Hill function to produce an oxygen EMT impact parameter decreasing from one in hypoxic conditions to approximately zero in oxygen-rich microenvironments, as shown in Fig. 18 (a). It has been observed experimentally that mesenchymal cells appear to promote EMT^111^. Here, we incorporate this using bystander signals and is modelled with a PDE. Cells higher on the N-cadherin rating scale secrete the chemical signal responsible for the bystander effect at increased rates. This leads to higher signal concentrations around mesenchymal cells’ location due to the low diffusion coefficient (1.0 microns^2^/min). This creates a chain reaction of EMT within the cells and is responsible for the clumps observed in the OVCAR-3 spheroids seen throughout the in-vitro experiments. Figure 18 (b) shows the quantitative impact that the signal concentration in the microenvironment has on the signal EMT impact parameter ranging between zero and one with a half max reached when the signal concentration is 3 units.

These oxygen and signal EMT impact parameters are viewed as the unweighted probability that an EMT jump will occur as a result of oxygen and signal concentrations respectively. Therefore, the values range between zero (highly unlikely) to one (highly likely). A cell line-dependent weighting for these terms is later added in Sections “OVCAR-3: Impact of microenvironment” and “SKOV-3: Impact of microenvironment”.

#### Impact of intra-cellular conditions

**Fig. 19:**
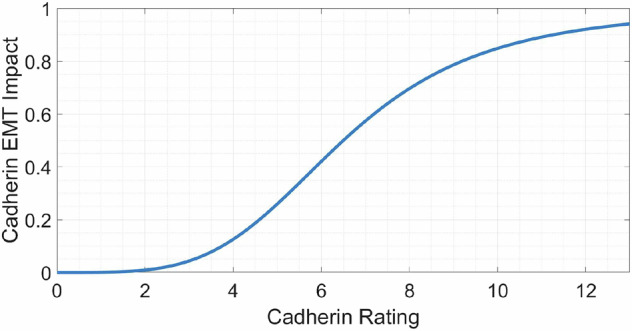
Cadherin EMT impact parameter, ${c}_{e}^{* }$, vs current N-cadherin rating. This shows how the current N-cadherin rating of a cell affects the likelihood of further steps up the N-cadherin rating. This positive correlation leads to increased stability in rating at either end of the EMT scale, as epithelial cells are less likely to undergo EMT on each iteration of the simulation than mesenchymal cells that are in otherwise identical conditions.

Cells are also given a cadherin EMT impact parameter, ${c}_{e}^{* }$, causing cells with a higher N-cadherin rating to progress faster through the EMT scale. Biologically, epithelial and mesenchymal cells exhibit the most stability, while hybrid cells displaying both epithelial and mesenchymal traits are considered metastable with the highest plasticity, retaining proliferative potential while also being migratory and invasive^112,113^. We introduce this phenomenon into the model using a Hill function based on the current rating of a cell, as illustrated in Fig. 19. The parameter ranges from zero for purely epithelial cells to near one for purely mesenchymal. We combine this variable with the oxygen EMT impact, ${o}_{e}^{* }$, and signal EMT impact, ${s}_{e}^{* }$, described in Section “Impact of Inter-Cellular Conditions”. Equation (2) shows how these variables are combined to generate an overall jump probability depending on the cell line. This ensures that each relevant inter-cellular and intra-cellular condition contributes to this probability in a synergistic way.

Similar to the inter-cellular variables in Fig. 18, the cadherin EMT impact parameter is seen as the unweighted probability that an EMT jump will occur as a result of the current N-cadherin rating of the cell. The value is set to range between zero and one prior to the cell-dependent weighting. We denote ${c}_{e}^{* }$ as the cadherin EMT impact, ${o}_{e}^{* }$ the oxygen EMT impact, and ${s}_{e}^{* }$ the signal EMT impact on rates of EMT in cancer cells shown in Figs. 18 and 19. A weighting term is incorporated into these impact parameters, for which we assign an EMT impact factor parameter, *α*, to both cell lines. This parameter quantifies the tendency for the cells to undergo EMT on each iteration of the simulation, depending on whether the cells are taken from OVCAR-3 or SKOV-3 cell lines.

#### OVCAR-3: Impact of microenvironment

Since EMT does not occur in the majority of OVCAR-3 cells during experimental observations, OVCAR-3 is given a low EMT impact factor of *α* = 1, as shown in Table 5. Larger values of *α*, such as that used in Table 6 for SKOV-3 cells, lead to larger weightings of the parameters when generating the jump probability term, *p*, in Equation (2). We analyse the influence of the value of this *α* parameter in the Supplementary Materials (SM). The cadherin EMT impact parameter has a low weighting for OVCAR-3 cells to ensure the number of clumps arising in the tumour throughout the 4-day simulation is not unrealistically high compared to biological observations (Fig. 1). This low weighting is set to be 0.001, based on trial simulations. The impact of oxygen is set to be medium, as hypoxia is not seen as a requirement for EMT but does act as a key catalyst for the process^75,114^. For simplicity, the weighting of the oxygen EMT impact is set to be 0.002, double that used for the cadherin EMT impact. The chemical signal impact responsible for the bystander effect has a large weighting to ensure disjoint clumps can be formed quickly despite the low diffusion of the signal. By observing simulations with different values of this weighting parameter, 0.01 is sufficiently high enough to allow clumps to appear within the time frame while avoiding a chain reaction of EMT and mesenchymal cells taking over the tumour.

**Table 5:** Values of the different variables used for the EMT probability in OVCAR-3 cells

Parameter	Unweighted	Parameter	Weighted
Name	Parameter Symbol	Weight (*α* = 1)	Parameter Symbol
Cadherin EMT Impact	${c}_{e}^{* }$	0.001	${c}_{e}=0.001\cdot {c}_{e}^{* }$
Oxygen EMT Impact	${o}_{e}^{* }$	0.002	${o}_{e}=0.002\cdot {o}_{e}^{* }$
Signal EMT Impact	${s}_{e}^{* }$	0.01	${s}_{e}=0.01\cdot {s}_{e}^{* }$

The signal EMT impact parameter has a large weighting to ensure sufficient levels of signal concentration can induce mesenchymal clump formation. The cadherin EMT impact parameter has a small weighting to ensure EMT does not occur too frequently within the tumour leading to a scenario in which the mesenchymal clumps begin to connect. OVCAR-3 has a low EMT impact factor to prevent excessive EMT from occurring throughout the simulation.

**Table 6:** Values of the different variables used for the EMT probability in SKOV-3 cells

Parameter	Unweighted	Parameter	Weighted
Name	Parameter Symbol	Weight (*α* = 5)	Parameter Symbol
Cadherin EMT Impact	${c}_{e}^{* }$	0.005	${c}_{e}=0.005\cdot {c}_{e}^{* }$
Oxygen EMT Impact	${o}_{e}^{* }$	0.01	${o}_{e}=0.01\cdot {o}_{e}^{* }$
Signal EMT Impact	${s}_{e}^{* }$	0.05	${s}_{e}=0.05\cdot {s}_{e}^{* }$

All parameters have an increased weighting to those used for OVCAR-3 cells to ensure sufficient EMT can occur to generate the pool of mesenchymal cells within the tumour interior. The EMT factor is increased from one in OVCAR-3 cells to five in SKOV-3 cells, as shown in the table.

#### SKOV-3: Impact of microenvironment

Unlike OVCAR-3 tumours, in SKOV-3 spheroids the red mesenchymal clumps are no longer distinguishable and instead a large pool covering the entire centre of the SKOV-3 tumour is formed, as shown in Fig. 1 (b). To ensure sufficient amounts of EMT occur to encapsulate this effect, the jump probability weightings are increased by a factor of five to that implemented for OVCAR-3 cells, as shown in Table 6. The jump probability is generated as shown in Equation (2) to ensure the biological observations remain in agreement with the simulation outputs. The shell of epithelial cells around the exterior of the tumour, as seen in experiments, is implemented by including an oxygen-dependent condition on the SKOV-3 cells. Hypoxic conditions in SKOV-3 tumours have been shown to upregulate the chemokine receptor CCR7, in turn inducing EMT development^115^. When oxygen concentration increases above a threshold value (set to 2.8 units), it is assumed mesenchymal cells undergo instant MET and are assigned the N-cadherin rating value of zero. This occurs only around the exterior of the tumour where oxygen is sufficient enough to cross this threshold. Table 6 shows the weightings of each parameter involved in generating the EMT probability for SKOV-3 cells. These increased weightings compared to those used in Table 5 for OVCAR-3 cells lead to vastly increased amounts of EMT. The EMT impact factor parameter is given a value of five for SKOV-3 cells, meaning the parameter weights are five times larger in Table 6 than in Table 5. This value ensures sufficient EMT occurs throughout the SKOV-3 tumour to allow the pool of interior mesenchymal cells to develop inside the tumour. Supplementary Fig. 5 shows how the final appearance of SKOV-3 tumours changes according to the value of the *α* term. From testing simulations with different values of this term, we find that setting *α* equal to five completely removes epithelial cells from the tumour interior after 4 simulated days.

### Modelling mesenchymal to epithelial transition

The effects of MET are explored in Section “Impact of MET and Initial Conditions”, with EMT and MET occurring in tumours simultaneously. To model this, various intra-cellular and inter-cellular conditions can affect the rate of MET. Hyperoxic conditions have been found to increase the conversion of EMT into MET within cells^116^. Biological observations also suggest hybrid cells possess the highest cell plasticity on the epithelial-mesenchymal scale^112^. This suggests MET is more likely to occur in hybrid cells than the stable mesenchymal cells. To incorporate these dynamics, we denote two new variables, ${c}_{m}^{* }$ defined as $1-{c}_{e}^{* }$ and ${o}_{m}^{* }$ defined as $1-{o}_{e}^{* }$. These represent the contributions of higher oxygen levels and lower cell N-cadherin rating to an increased probability of MET on each iteration. In terms of probability, these can be seen as the compliments of ${c}_{e}^{* }$ and ${o}_{e}^{* }$ respectively. These parameters are shown in Table 7 and applied to Equation (3) to calculate the jump-down probability on each iteration. For simplicity, we use the same “jump down” probabilities for the two cell lines, using similar values and weightings to those in Table 6 to account for the lack of signal impact involved in the EMT dynamics.

**Table 7:** Weightings for parameters used in generating the rates of MET for cells

Parameter	Unweighted	Parameter	Weighted
Name	Parameter Symbol	Weight	Parameter Symbol
Cadherin MET Impact	${c}_{m}^{* }$	0.005	${c}_{m}=0.005\cdot {c}_{m}^{* }$
Oxygen MET Impact	${o}_{m}^{* }$	0.01	${o}_{m}=0.01\cdot {o}_{m}^{* }$

Only the current N-cadherin rating and the oxygen levels around the cell change the rate at which a cell can move down the cadherin level.

### Experimental methods

Experimental data presented in this model uses EMT marker profiles of SKOV-3 and OVCAR-3 cell populations grown as 2D monolayers and 3D spheroids (10.1186/s12964-024-01806-4). Differences between profiles of 2 and 3D cultured populations were determined via RT-qPCR, immunoblots, and immunofluorescence. For protein analysis, immunoblotting was performed using primary antibodies against E-cadherin (clone G10, Santa Cruz Biotech) and N-cadherin (clone 13A9, Santa Cruz Biotech), with GAPDH (clone O411, Santa Cruz Biotech) used as a loading control for normalization. For fluorescence analysis of tumour spheroids, immunostaining was carried out as previously described by us (10.1186/s12964-024-01806-4). In brief, an initial number of cells were cultured as spheroids using the hanging drop method and subsequently fixed in 4% PFA with 1% Triton in PBS for 3 h at 4 ^∘^C. After washing with PBS, spheroids underwent a dehydration and rehydration process through sequential exposure to increasing methanol concentrations (25%, 50%, 75%, and 95%) for 30 min each, followed by 100% methanol for 5 h. The rehydration was achieved by reversing the methanol gradient back to 0% methanol and 100% PBS. Spheroids were blocked overnight at 4 ^∘^C in PBST containing 3% BSA, followed by incubation with primary antibodies for E-cadherin (clone: EP700Y, Abcam) and N-cadherin (clone: 13A9, Santa Cruz Biotech) and fluorescent secondary antibodies (Alexa Fluor® 555-conjugated polyclonal goat anti-rabbit antibody (ab150086, Abcam) and Alexa Fluor® 488-conjugated polyclonal goat anti-mouse antibody (ab150117, Abcam). Before imaging, spheroids were stained with Hoechst and mounted onto microscope slides for visualisation.

## Supplementary information

Supplementary Materials

## Data Availability

Biological observations and results are available for public use and can be found at 10.1186/s12964-024-01806-4^85^. Anonymised data sets used in the study are available from the authors upon request. Parameters used from model projects of Physicell are available for public use and can be found in the Physicell software at 10.1371/journal.pcbi.1005991 in the example template project^69^.

## Code availability

The code used for this model is available on GitHub and can be found via the following link: https://github.com/Sam-M-Oliver/Role-of-EMT-Public.
