# Supplementary material for: Exploring the role of EMT in ovarian cancer progression using a multiscale mathematical model
Source: NPJ Syst Biol Appl. 2025 Apr 17;11:36. doi: 10.1038/s41540-025-00508-y (PMC12006308; doi:10.1038/s41540-025-00508-y)
Supplement: Supplementary file 1 — Supplementary Materials [file 41540_2025_508_MOESM1_ESM.pdf]

Supplementary Materials: Exploring the role of EMT in Ovarian Cancer Progression: Insights from a multiscale mathematical model

Samuel Oliver[1], Michael Williams[2], Mohit Kumar Jolly[3], Deyarina Gonzalez[2], and Gibin Powathil[1]

[1]Department of Mathematics, Swansea University, Swansea, United Kingdom
[2]Department of Biomedical Sciences, Swansea University, Swansea, United Kingdom
[3]Department of Bioengineering, Indian Institute of Science, Bangalore, India

March 28, 2025

Supplementary Section 1 Sensitivity Analysis

By utilising an adaptation of Latin Hypercube Sampling, global sensitivity analysis can be performed on the model [1]. Analysis is performed on six parameters: c_c (cadherin cycling impact), o_c (oxygen cycling impact), and p_c (pressure cycling impact) used to calculate the cell cycling rate in Equation 1, as well as c_e (cadherin EMT impact), o_e (oxygen EMT impact), and s_e (signal EMT impact) used to calculate the EMT jump probability in Equation 2. The values of each parameter is ranged by $\pm 20\%$ using 51 equally spaced values for each parameter. Each of these 51 values for each parameter is then assigned to one of the 51 simulations we run, resulting in a unique value for each parameter across the simulations. Two output variables from each simulation following 96 hours of simulated time are investigated: the total cell population in the tumour and the fraction of the tumour considered as mesenchymal. Here the classification of epithelial and mesenchymal cells is made according to if the cadherin rating is 0-6 (epithelial) or 7-13 (mesenchymal). This selection is due to the importance of these results in cancer diagnosis. The stage a patient is deemed to be at depends largely upon the size and metastatic ability of the tumour. These outputs are then compared with the input parameter values for each of the six parameters of interest, with the Pearson Product Correlation (PCC) value calculated to find the nature and magnitude of the correlation between the ith input x_i and output y_i (See Supplementary Equation 1) [2]. The value of this PCC variable shows if the correlation between the input parameter and the simulation output is weak, moderate, or strong (See Supplementary Table 1).

$$PCC = \frac{\sum_{i=1}^{n}\big((x_i - \bar{x})(y_i - \bar{y})\big)}{\sqrt{\sum_{i=1}^{n}(x_i - \bar{x})^2 (y_i - \bar{y})^2}}. \qquad \text{(Supplementary Equation 1)}$$

Coefficient Magnitude	Strength of Correlation
0	No Correlation
Up to 0.4	Weak Correlation
0.4 up to 0.7	Moderate Correlation
Over 0.7	Strong Correlation
1	Perfect Correlation

Supplementary Table 1: Evaluations of different values for the PCC. Positive/negative values suggest a likely positive/negative correlation [3]. Stronger correlations between the input and output result in higher magnitudes of the coefficient.

Supplementary Section 1.1 Total OVCAR-3 Population

Figure 1 shows the relationship between the key model parameters highlighted previously and the total population of OVCAR-3 cells after 96 hours of simulated time. The maximum cadherin cycling impact (a) has a moderate influence and the maximum oxygen cycling impact (b) has a strong influence on the final population size respectively. The maximum pressure cycling impact (c) is found to have a statistically weak influence on the final cell population, suggesting small variations in this value do not have a major impact on the population size in the tumour. The three parameters used in calculating the jump probability (Equation 2) all have a weak correlation to the total population of OVCAR-3 cells. This is to be expected since these parameters have no direct link to the cycling rate of the cell, instead only affecting the cadherin rating in the tumour.

Supplementary Section 1.2 Mesenchymal Fraction of OVCAR-3 Tumours

Figure 2 shows the relationship between the six parameters and the fraction of the total tumour population considered mesenchymal after 96 hours of simulated time. The oxygen EMT impact (e) and signal EMT impact (f) weights have a strong and moderate influence on the final composition of the OVCAR-3 tumours respectively. The cadherin EMT impact weight is the only weighting in Table 5 with a weak correlation to the final tumour composition. This is likely due to the small weighting that was assigned to the cadherin EMT impact used in Equation 2. All other input parameters including those used in Table 3 have a negligible impact on tumour composition, suggesting there is a reasonable level of stability with respect to the user parameters involved in the cell cycling rate (Equation 1).

The correlation between parameters and the outputs between Sections Supplementary Section 1.1 and Supplementary Section 1.2 show mostly opposite trends. Strong correlations between parameters in Section Supplementary Section 1.1 generally show weak correlations in Section Supplementary Section 1.2 and vice versa. This is due to the fact that these parameters either link directly to the cell cycling rate or the EMT probability, rather than both.

Supplementary Section 1.3 Total SKOV-3 Population

Figure 3 shows the relationship between the same six parameters highlighted previously and the total population of SKOV-3 cells. The maximum cadherin cycling impact (a) and oxygen cycling impact (b) have a moderate and strong influence on the final population size respectively. Similar

to OVCAR-3 tumours, the maximum pressure cycling impact has a weak correlation to the final population of SKOV-3 tumours, along with all the EMT impact weight parameters.

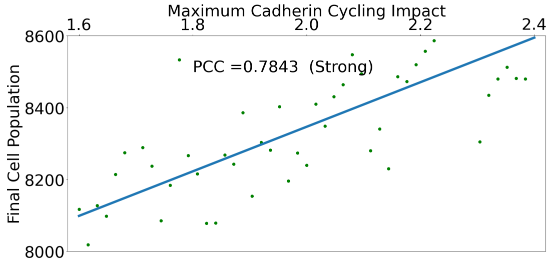

(a) Cadherin cycling impact parameter

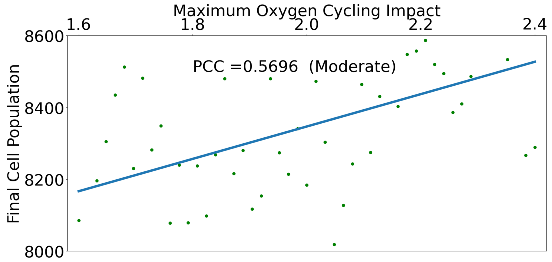

(b) Oxygen cycling impact parameter

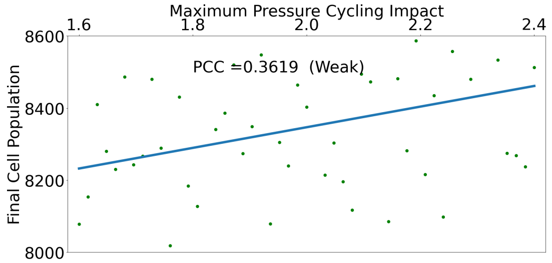

(c) Pressure cycling impact parameter

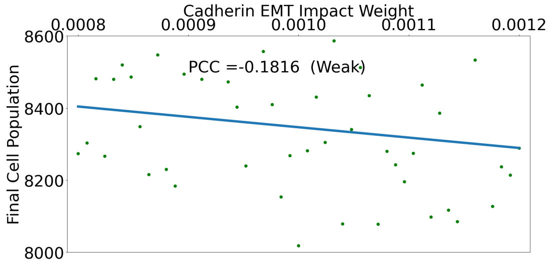

(d) Cadherin EMT impact parameter

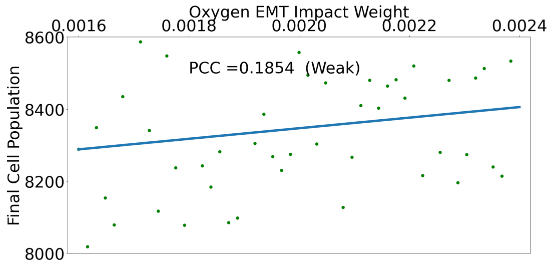

(e) Oxygen EMT impact parameter

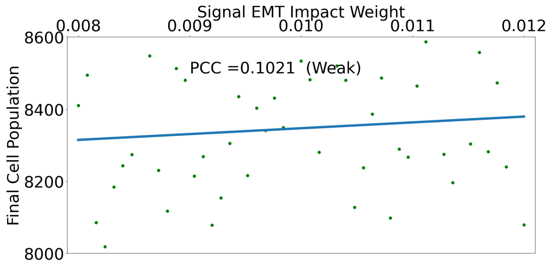

(f) Signal EMT impact parameter

Supplementary Figure 1: **The impact of parameter values on the total cell population in OVCAR-3 tumours.** The total tumour cell population after four days is found and compared for various parameter values. The PPC is given for each parameter across 51 simulations. Figures (a), (b), and (c) concern the cycling rate used in Equation 1, while Figures (d), (e), and (f) concern rates at which EMT can occur within the cells used in the jump probability in Equation 2.

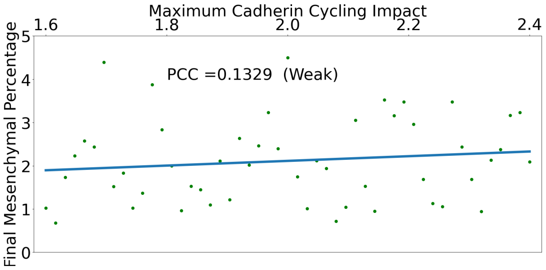

(a) Cadherin cycling impact parameter

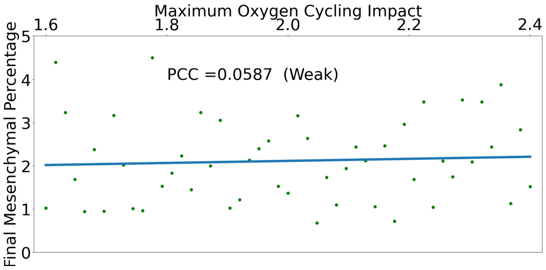

(b) Oxygen cycling impact parameter

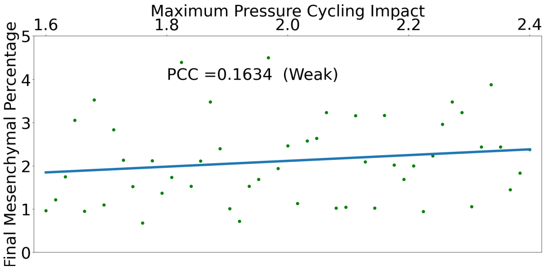

(c) Pressure cycling impact parameter

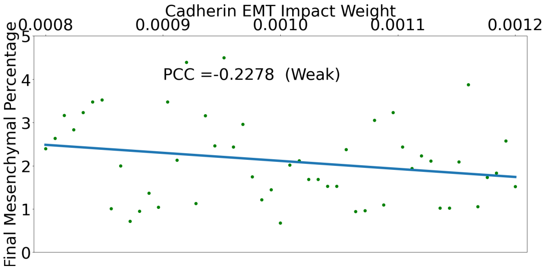

(d) Cadherin EMT impact parameter

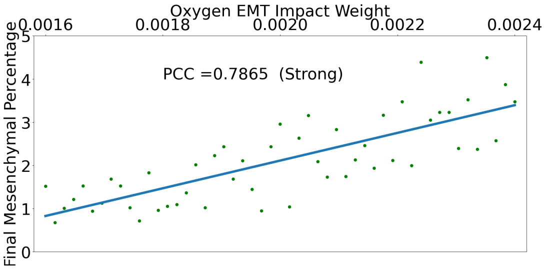

(e) Oxygen EMT impact parameter

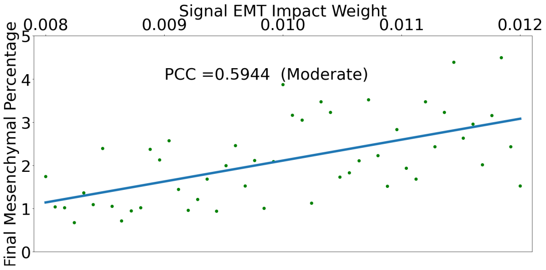

(f) EMT signal impact parameter

Supplementary Figure 2: **The impact of parameter values on the final composition of the OVCAR-3 tumour.** The fraction of the total tumour cell population classed as mesenchymal is found and compared for various parameter values. The PPC is given for each parameter across 51 simulations. Figures (a), (b), and (c) concern the cycling rate used in Equation 1, while Figures (d), (e), and (f) concern rates at which EMT can occur within the cells used in the jump probability in Equation 2.

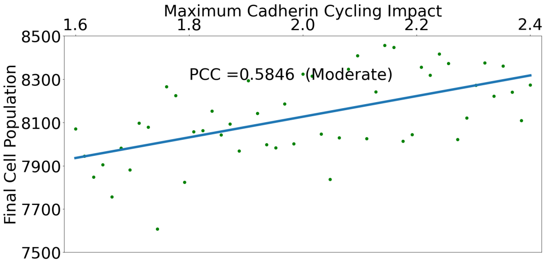

(a) Cadherin cycling impact parameter

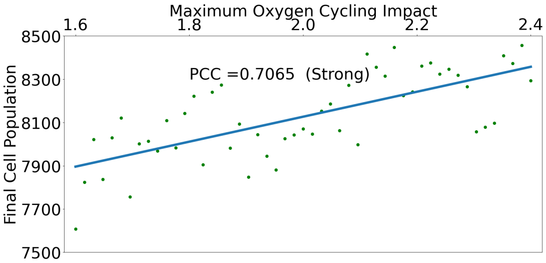

(b) Oxygen cycling impact parameter

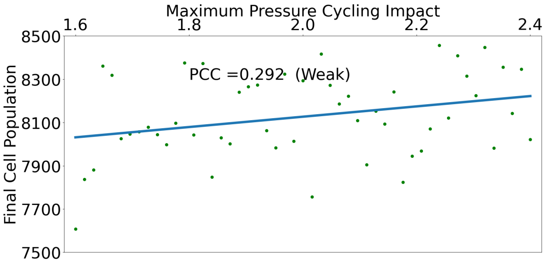

(c) Pressure cycling impact parameter

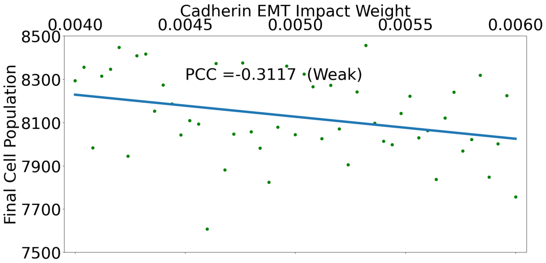

(d) Cadherin EMT impact parameter

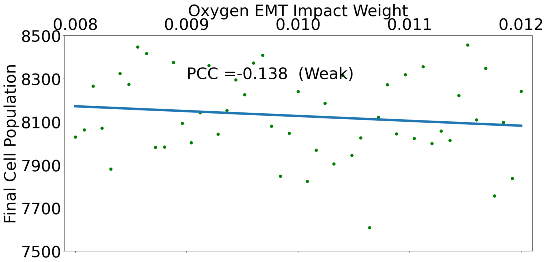

(e) Oxygen EMT impact parameter

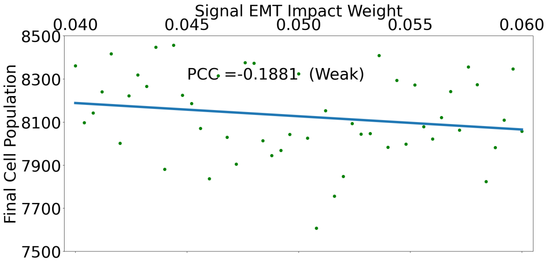

(f) Signal EMT impact parameter

Supplementary Figure 3: **Impact of parameter values on the final total cell population in the SKOV-3 tumour.** The total tumour cell population after four days is found and compared for various parameter values. The PPC is given for each parameter across 51 simulations. Figures (a), (b), and (c) concern the cycling rate used in Equation 1, while Figures (d), (e), and (f) concern rates at which EMT can occur within the cells used in the jump probability in Equation 2.

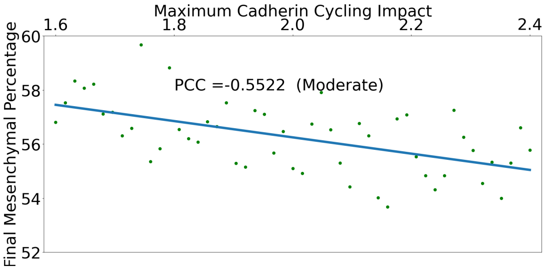

(a) Cadherin cycling impact parameter

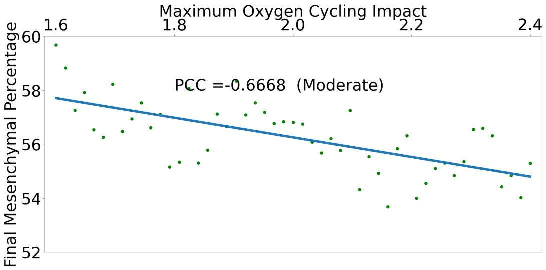

(b) Oxygen cycling impact parameter

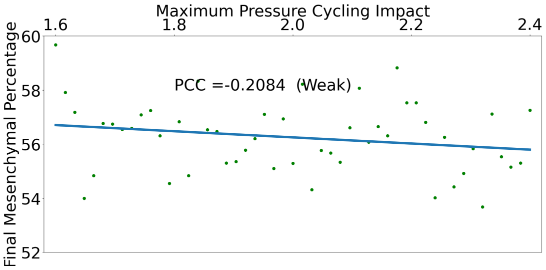

(c) Pressure cycling impact parameter

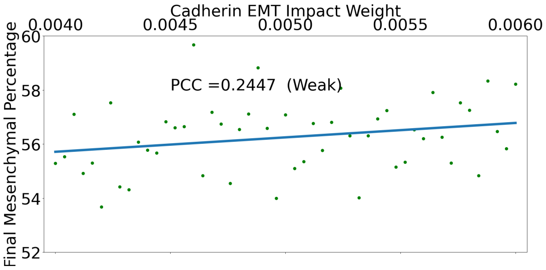

(d) Cadherin EMT impact parameter

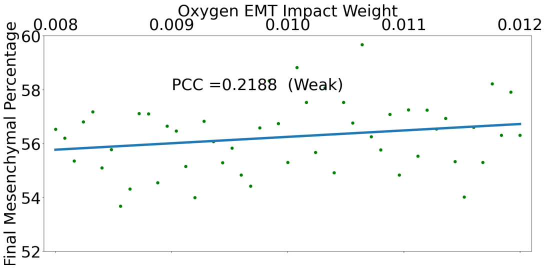

(e) Oxygen EMT impact parameter

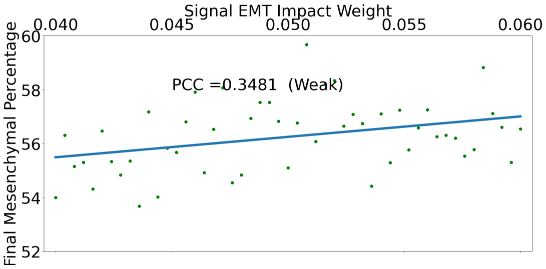

(f) EMT signal impact parameter

Supplementary Figure 4: **Impact of parameter values on the final composition of the SKOV-3 tumour.** The fraction of the total tumour cell population classed as mesenchymal is found and compared for various parameter values. The PPC is given for each parameter across 51 simulations. Figures (a), (b), and (c) concern the cycling rate used in Equation 1, while Figures (d), (e), and (f) concern rates at which EMT can occur within the cells used in the jump probability in Equation 2.

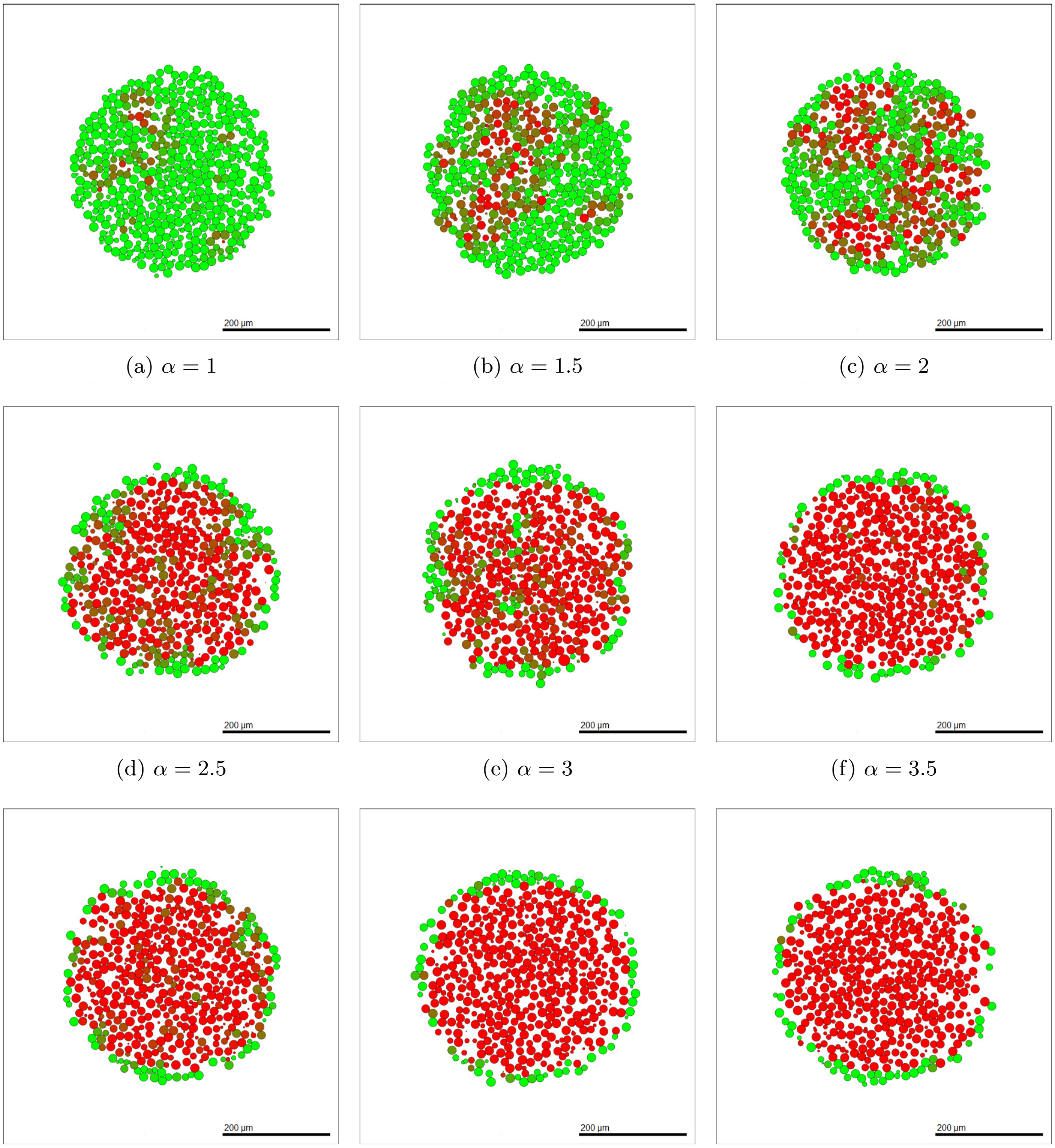

(a) $\alpha = 1$ (b) $\alpha = 1.5$ (c) $\alpha = 2$

(d) $\alpha = 2.5$ (e) $\alpha = 3$ (f) $\alpha = 3.5$

(g) $\alpha = 4$ (h) $\alpha = 4.5$ (i) $\alpha = 5$

Supplementary Figure 5: **Impact of the value of α on the final composition of the SKOV-3 tumour.** Larger values of this α term lead to increased weightings for the parameters used in the EMT jump probability in Equation 2. Snapshots of the $z = 0$ plane are taken after four days of simulated time and compared for different α values.

Supplementary Section 1.4 Mesenchymal Fraction of SKOV-3 Tumours

Figure 4 shows the relationship between the six parameters and the fraction of the SKOV-3 tumour population considered mesenchymal after 96 hours of simulated time. Surprisingly here, no EMT impact weight parameter has more than a weak impact on the percentage of mesenchymal cells in the final tumour, despite their direct link to the EMT jump probability (Equation 2). This is due to the fact that the interior SKOV-3 cells complete EMT and become mesenchymal regardless of small changes in these parameters. As well as this, the maximum cadherin cycling impact and oxygen cycling impact both have moderately negative correlations to the mesenchymal fraction of cells in the tumour. Increased cycling rates allow the tumour to grow faster, allowing the exterior cells to approach the domain boundaries. Here, the oxygen available to the cells is higher due to the Dirichlet boundary conditions imposed, allowing a thicker shell of epithelial cells, thus decreasing the fraction of the tumour cells considered to be mesenchymal.

Supplementary Section 1.5 Cell Line Differentiation

Another approach to sensitivity analysis can be to change the difference in weighting terms between Tables 5 and 6. In these tables the weightings of parameters used in the EMT jump probability, p, in Equation 2 have five times larger weightings for SKOV-3 cells than OVCAR-3 cells. This creates the clearly distinguishable difference in tumour layouts, with OVCAR-3 possessing disjoint clumps of mesenchymal cells while SKOV-3 tumours have a pool of mesenchymal cells making up the entire interior. We define α to denote the factor at which SKOV-3 cells have a larger weighting than that used for OVCAR-3 cells in Table 5. For example, $\alpha = 5$ in Table 6. We can vary the value of α to explore at which point the tumour shows OVCAR-3 and SKOV-3 characteristics. Figure 5 shows the tumour appearance after 96 hours of simulated time for different values of α.

In Figure 5 (b) and (c) where $\alpha = 1.5$ and $\alpha = 2$, the tumour appears to show a hybrid state of OVCAR-3 and SKOV-3. Large clumps of mesenchymal cells are formed, however, due to their increased size, these begin to overlap and make up a large proportion of the inside of the tumour. With values of α between two and four in Figures 5 (d), (e), and (f), the pool of mesenchymal cells has formed with only occasional epithelial cells appearing within the tumour interior. This appears closer to the original SKOV-3 tumour appearance. The slow transition between the cell lines across the figures display that the switch in the model between SKOV-3 and OVCAR-3 cells can be continual and non binary, generating cells with characteristics of both cell lines.

Supplementary Section 2 Model Parameters

Parameter	Units	Value
S Phase Duration	min	480 [4]
G2 Phase Duration	min	240 [4]
M Phase Duration	min	60 [4]
Apoptosis Rate	1/min	1e-5
Total Cell Volume	$micron^3$	2494
Nuclear Cell Volume	$micron^3$	540
Cell-Cell Repulsion Strength	micron/min	10
Oxygen Uptake Rate	1/min	0.6
Signal Uptake Rate	1/min	0

Supplementary Table 2: **Cellular Parameter Values.**

Parameter	Units	Oxygen	Signal
Diffusion Coefficient	$micron^2/min$	1e5	0
Decay Rate	1/min	10	1
Dirichlet Boundary Conditions	dimensionless	38	0

Supplementary Table 3: **Substrate Parameter Values.**

Supplementary References

[1] S. Hamis, S. Stratiev, and G. G. Powathil, "Uncertainty and sensitivity analyses methods for agent-based mathematical models: An introductory review," *The Physics of Cancer: Research Advances*, pp. 1–37, 2021.

[2] M.-T. Puth, M. Neuhäuser, and G. D. Ruxton, "Effective use of pearson's product–moment correlation coefficient," *Animal behaviour*, vol. 93, pp. 183–189, 2014.

[3] C. P. Dancey and J. Reidy, *Statistics without maths for psychology.* Pearson education, 2007.

[4] R. M. Bavle, "Mitosis at a glance," *Journal of Oral and Maxillofacial Pathology*, vol. 18, no. Suppl 1, pp. S2–S5, 2014.
